# Hydride and Seek: Comparing Crystallographic Hydride Placement Techniques with an Open-Shell Cobalt Complex

**DOI:** 10.1021/acscentsci.6c00632

**Published:** 2026-06-23

**Authors:** Ryan S. Donnelly, Theodore J. Gerard, Sebastian M. Krajewski, Brandon Q. Mercado, Xiaoping Wang, Patrick L. Holland

**Affiliations:** † Department of Chemistry, 5755 Yale University, New Haven, Connecticut 06520, United States; ‡ Neutron Scattering Division, 6146 Oak Ridge National Laboratory, Oak Ridge, Tennessee 37831, United States

## Abstract

Locating hydrides is crucial in organometallic chemistry but difficult to do accurately using X-ray diffraction. Electron diffraction has been proposed as a way to overcome this problem but has not been systematically compared to neutron diffraction and to quantum crystallography (Hirshfeld atom refinement, HAR) to test this hypothesis. Here, we present a comparative analysis of methods for a terminal cobalt hydride complex by comparing a single-crystal neutron diffraction reference structure to results from single-crystal X-ray diffraction with and without Hirshfeld atom refinement (HAR, NoSpherA2), density functional theory (DFT), and electron diffraction (3D-ED/MicroED) refined under kinematical and dynamical formalisms. Conventional X-ray diffraction gives lower precision than neutron diffraction as expected. Despite expected improvements, HAR gives systematic deviation from the neutron benchmark. Interestingly, optimized DFT equilibrium geometries are closer to the neutron value than the value from HAR. On the other hand, electron diffraction with a high-quality data set coupled with dynamical refinement localizes the hydride in difference maps and gives excellent agreement with the neutron data. Dynamical refinement is crucial, as kinematical refinement does not allow assignment of a hydride peak. This cross-modal comparison defines the conditions under which 3D-ED/MicroED delivers high-precision metal–hydride distances for this open-shell cobalt hydride.

## Graphical abstract

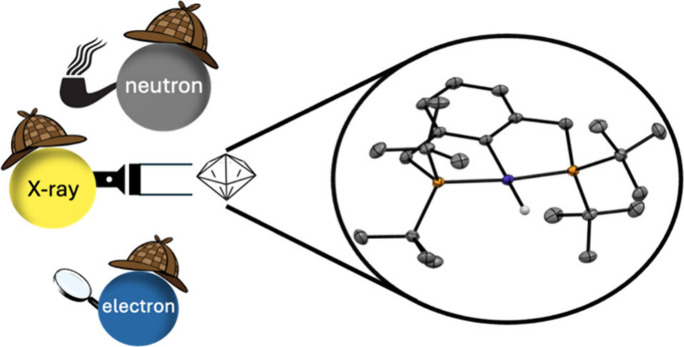

## Introduction

Hydride complexes, those containing a M–H bond, have played a crucial role in organometallic chemistry for decades because of their high reactivity and their intermediacy in many catalytic reactions.^1–4^ However, a persistent and recurring problem with characterizing hydride complexes with single-crystal X-ray diffraction (XRD) is the difficulty of accurately locating the metal-bound hydrogen atom. Due to the weak scattering of hydrogen (Figure 1a), the peaks for hydrides in electron density maps frequently are indistinguishable from noise and Fourier truncation artifacts. This issue can be magnified in paramagnetic hydride complexes because it is difficult to corroborate their presence using ^1^H NMR spectroscopy.^5^ Another issue is that the commonly used atomic form factors assume a spherical distribution of electron density around each atom (Independent Atom Model, IAM),^6^ but chemical bonding leads to aspherical electron distributions. Since hydrogen atoms have one valence electron and no core electrons, this effect is particularly pronounced and leads to systematic errors from underestimated X–H distances.^7,8^

**Figure 1:**
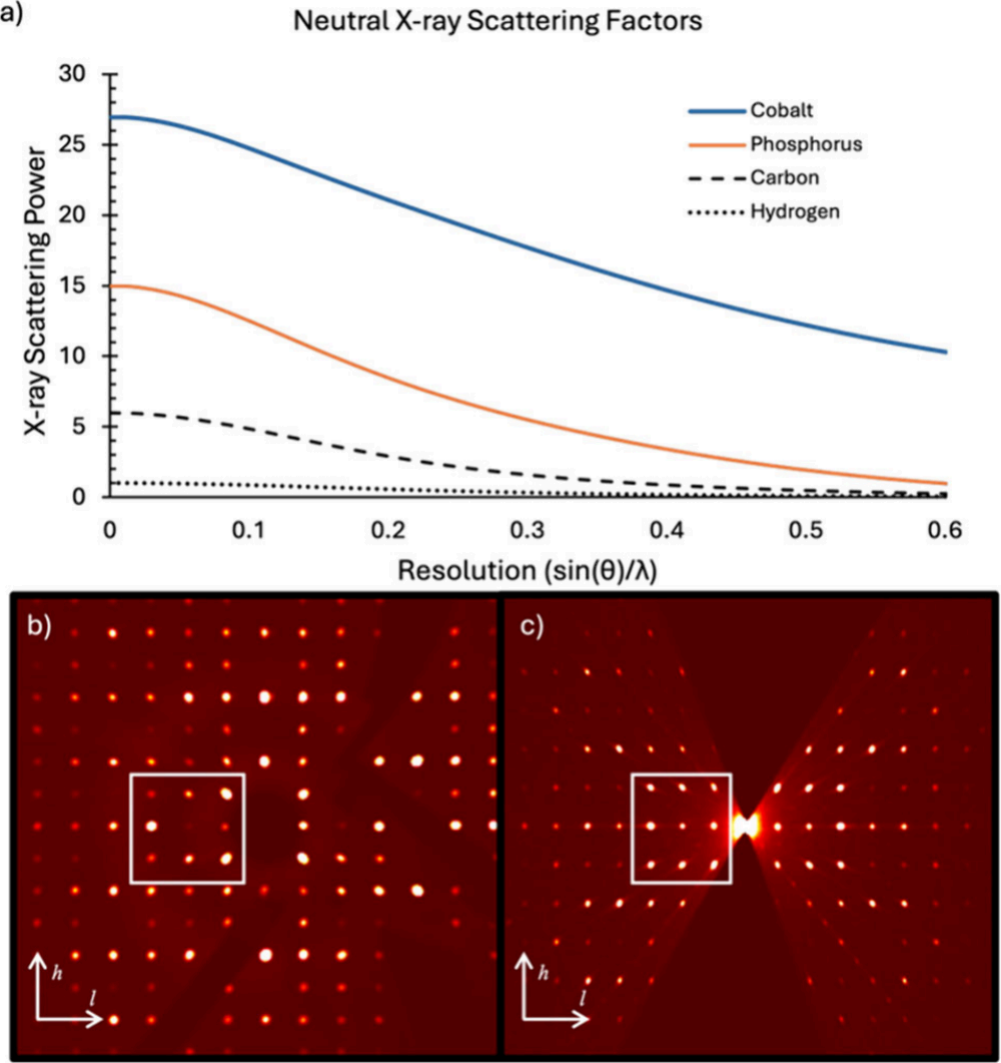
a) Neutral X-ray scattering factors for four representative elements.^8–11^ b) Kinematical diffraction pattern from X-ray diffraction oriented in the *h*0*l* plane. The white box indicates a set of nine reflections with independent intensities. c) Dynamical diffraction pattern from electron diffraction oriented in the *h*0*l* plane. The white box indicates the same set of reflections from (b). Note how all nine reflections now appear to be of similar intensity.

This has led to efforts to create aspherical electron density models, such as the multipole model.^12–20^ The best known is Hirshfeld Atom Refinement (HAR),^21^ which starts with atomic coordinates derived from XRD and performs molecular wavefunction calculations to derive an electron density distribution.^22^ HAR has been applied with full anisotropic treatment of hydrogen atoms to inorganic structures,^23^ small organic molecules, and disordered structures.^24^ However, even with these enhancements to XRD refinement and modeling, X-rays are still fundamentally ill-suited to detect H atoms precisely, particularly near heavy atoms in hydride complexes.

Neutron diffraction is more effective for detecting hydride H atoms because neutrons are scattered by nuclei, and the scattering does not depend on the number of electrons. Furthermore, hydrogen has a distinctive negative scattering length of –3.741 fm, which allows H atoms to be located easily from a Fourier difference map.^25^ Through neutron diffraction, accurate atomic positions and anisotropic displacement parameters (ADPs) for hydrogens can be obtained.^26^ However, neutron diffraction requires access to modern high-performance single-crystal neutron diffractometers available in specialized user facilities.^27,28^ Even with these tools, neutron diffraction requires relatively large crystals, and therefore remains less routine than other diffraction methods.

More recently, electron diffraction (ED) has emerged as an alternative to XRD for the determination of structures using microcrystals of biological, organic, and organometallic compounds.^29–35^ In electron diffraction, the incident electron beam is scattered through Coulombic interactions with both the nucleus and electrons in a crystalline sample. As a result, electron diffraction can more easily localize lighter elements in the presence of heavy atoms. This advantage of electron diffraction, though noted in the literature,^36–39^ has not been recognized as much as the advantages of using micron-sized crystals.^29,31^

A complication is that the kinematical scattering model used in XRD (Figure 1b) has limited application to ED since electrons interact more strongly with crystals than X-rays. As a result, one incident electron can undergo multiple scattering events (dynamical scattering).^30^ Because an electron has the potential to scatter multiple times, the relationship *I*_*hkl*_ ∝ |*F*_*hkl*_|^2^ is no longer valid, as intensities for a given set of reflections become dependent on each other (Figure 1c).^40^ To most accurately model ED data and fully realize the potential to find H atoms, refinement must consider dynamical scattering. Researchers have worked on methods for full dynamical refinement of electron diffraction data, but initial implementations were only applied to oriented diffraction patterns.^41^ Recently, Palatinus and co-workers have developed a more generalized application of dynamical refinement.^42–44^ When dynamical effects are modeled, hydrogen atoms can be localized as a result of electron diffraction’s inherent sensitivity to lighter elements. Indeed, recent studies have demonstrated the effectiveness of electron diffraction for locating hydrogen atoms in organic molecules^39,45–47^ and even bridging hydrides in a dinuclear zirconocene complex (Schwartz’s reagent).^48^

With the improvement of XRD modeling and ED techniques, efforts have been made to compare these methods to neutron data. For example, Woińska et al. compared HAR refined values of the X–H bond lengths in five inorganic crystal structures to distances refined with neutron data from the same compound (or closely related analogs).^49^ Similar comparisons have been made for electron diffraction data and neutron data with organic samples.^43,45,50^ Furthermore, prior studies have demonstrated the advantages of electron diffraction over X-ray diffraction for determining hydrogen positions in ice and O–H···O hydrogen-bond networks, including paramagnetic metal salt hydrates.^51,52^ The Woźniak group has even applied multipole modeling to electron diffraction data. However, to our knowledge, there is no study comparing all three diffraction techniques (electron, X-ray, neutron) for the same transition metal–hydride complex.

The present study evaluates whether 3D-ED/MicroED can provide more accurate M–H distances than routine XRD-based methods for a transition-metal hydride, where the challenge is to locate the light atom with high accuracy immediately adjacent to a heavier atom. We use a pincer-ligated cobalt­(II)–hydride complex, whose high crystallographic symmetry and ease of crystallization facilitate comprehensive crystallographic analysis of the Co–H bond length using XRD, HAR, neutron diffraction, and electron diffraction, with DFT optimization providing an additional computational reference point. By comparing all methods on the same chemical system, this study clarifies when each approach can deliver a quantitative metal−hydride bond length and identifies a simple protocol that can be used for other challenging transition-metal hydride complexes.

## Results

### Synthesis and Characterization of (^tBu^PCP)­CoH

The stoichiometric reaction between (^tBu^PCP)­CoBr (**CoBr**) and KHBEt_3_ in tetrahydrofuran (THF) under an argon atmosphere affords (^tBu^PCP)­CoH (**CoH**) in isolated yields around 70%. The ^1^H NMR spectrum of a solution in THF-*d*_8_ displays four paramagnetically broadened resonances between δ −13 and 36 ppm. To determine the spin ground state in **CoH**, we measured its dc magnetic susceptibility between 2 and 300 K under an applied magnetic field of 0.500 T. The χ_M_ *T* value for **CoH** between 10 and 300 K is 0.70 cm^3^·K/mol, which is somewhat high for an *S* = 1/2 ground state (spin-only value of 0.38), potentially arising from unquenched orbital angular momentum (Figure 2b). To test this hypothesis, we measured the X-band EPR spectrum of **CoH** in frozen toluene at 10 K (Figure 2c). The EPR spectrum was simulated as an *S* = 1/2 spin system with *g*_*x*,*y*,*z*_ = [4.10, 1.88, 1.40] and *A*(^59^Co, *I* = 7/2) = [1391, 270, 200] MHz. The parameters used to model the EPR spectrum can also be used to simulate the dc magnetic susceptibility data (see SI for further details). The data are consistent with similar previously reported cobalt­(II) complexes.^53–55^

**Figure 2:**
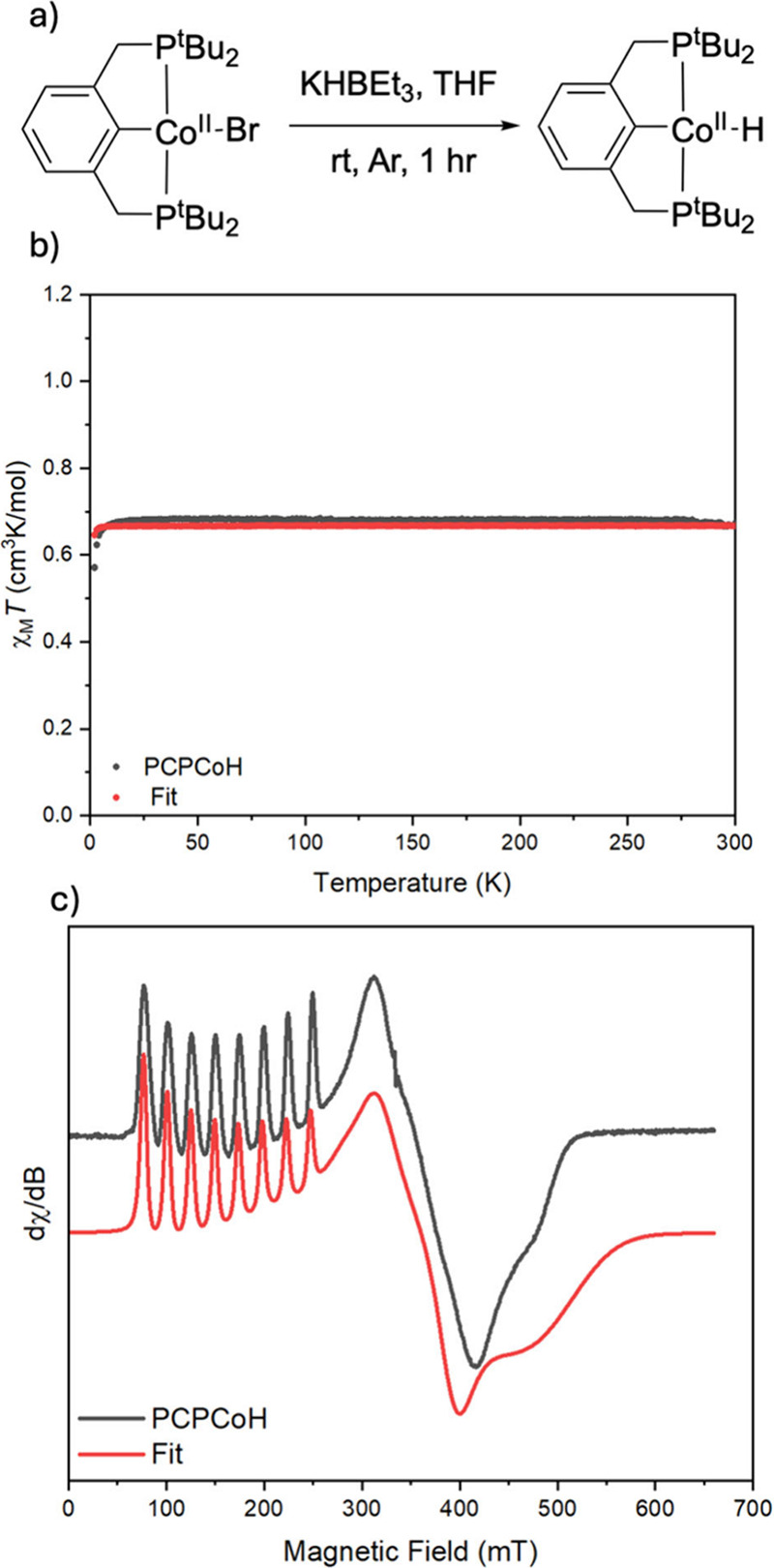
a) Synthetic scheme for the formation of **CoH**. b) Plot of dc magnetic susceptibility data for **CoH** under an applied magnetic field of 0.500 T. c) EPR spectrum of a frozen solution of **CoH** (5 mM toluene) collected at 9.352 GHz and 10 K.

### Neutron Diffraction

Single-crystal neutron diffraction data were collected at 100 K from a 1.18 × 0.88 × 0.12 mm single crystal of **CoH** on the TOPAZ beamline at the Spallation Neutron Source, Oak Ridge National Laboratory.^27^ An initial refinement was performed with anisotropic thermal parameters for all non-hydrogen atoms except for the hydride. The difference Fourier map of the nuclear scattering density showed a pronounced negative peak with a height (hole) of −6.3(2) fm Å^–3^ (Figure 3b). The negative sign is consistent with assignment to a hydrogen atom,^25^ and this site was included in subsequent structure refinement with anisotropic thermal parameters for all atoms. Structure solution and refinement to convergence were performed using SHELXL.^56^ The refined Co–H distance was found to be 1.54(2) Å (Figure 3).

**Figure 3:**
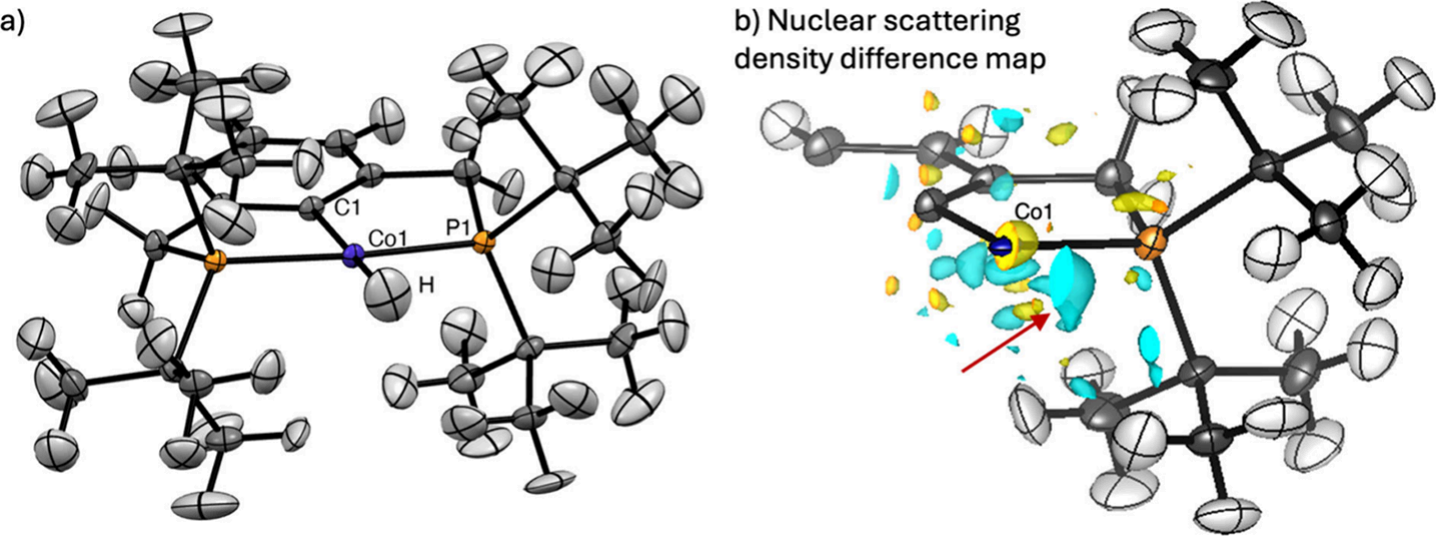
a) ORTEP plot of the neutron diffraction structure of **CoH**. All atoms are refined with anisotropic thermal parameters. Key atoms are labeled for clarity. Ellipsoids are drawn at the 50% probability level. b) Fourier difference map of the asymmetric unit from the neutron diffraction data (yellow indicates positive density and blue indicates negative). The arrow indicates the large, negative peak near the cobalt center Co1 diagnostic of a hydride.

### X-ray Crystallography Using IAM

Low-temperature diffraction data (ω-scans) of a crystal of **CoH** were collected on a Rigaku MicroMax-007HF diffractometer with Mo Kα radiation (λ = 0.71073 Å) to a resolution of 0.50 Å. Carbon-bound hydrogen atoms were included in the model at geometrically calculated positions and refined using a riding model. The crystal structure of **CoH** reveals a cobalt center ligated by two phosphorus atoms and the central carbon of the pincer ligand (Figure 4a). The Co–P bond lengths are 2.1653(2) Å, and the Co–C_aryl_ distance is 1.9627(14) Å. All of these distances are as expected for PC­(aryl)­P pincers ().^57^ The hydride can be tentatively identified as the largest difference peak (Figure 4b). Placing the hydride and refining with the IAM results in a Co–H distance of 1.49(7) Å.

**Figure 4:**
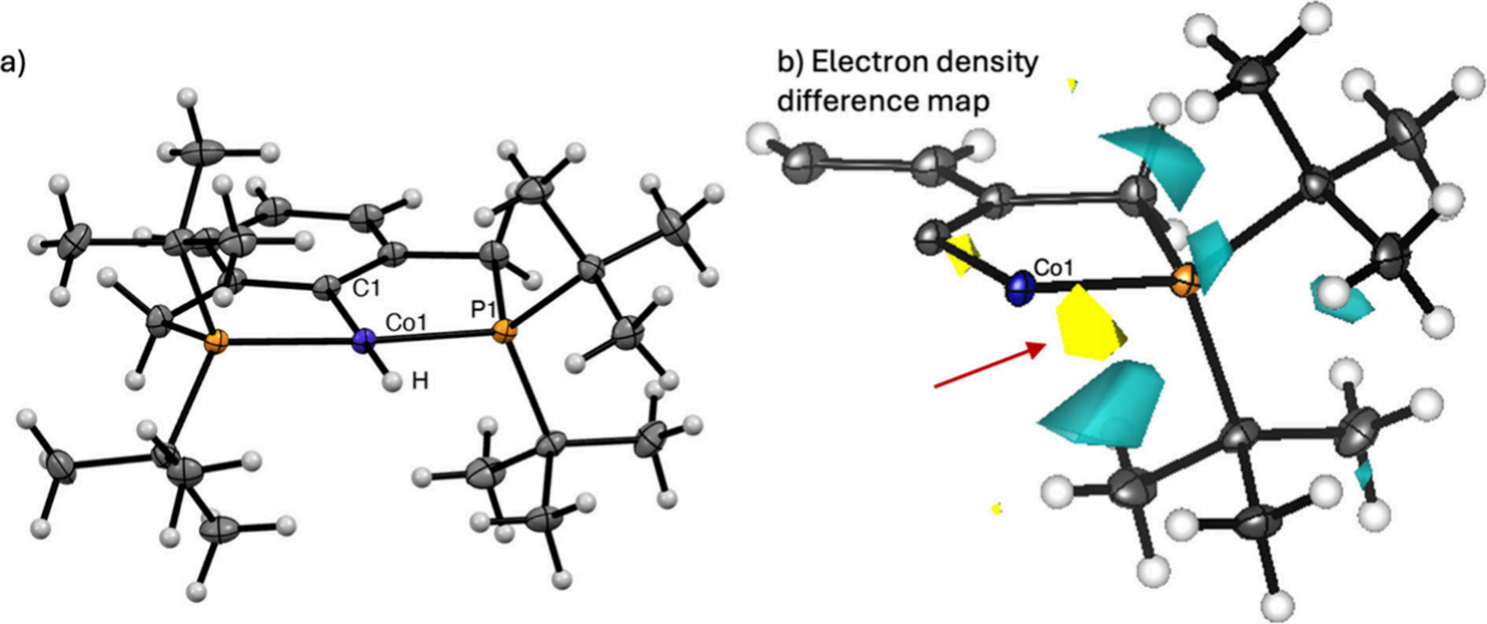
a) ORTEP plot of the X-ray diffraction structure of **CoH** using the Independent Atom Model. All non-hydrogen atoms are refined with anisotropic thermal parameters. Carbon-bound hydrogens use a riding model. Key atoms are labeled for clarity. Ellipsoids drawn at the 50% probability level. b) View of one asymmetric unit (half of the molecule) as the Fourier difference map (yellow indicates positive electron density while blue indicates negative). The arrow indicates the positive electron density near the cobalt center diagnostic of a hydride.

### Hirshfeld Atom Refinement with NoSpherA2

The high-resolution X-ray data collected as described above for **CoH** were then used for Hirshfeld atom refinement. After solving and refining the structure with the IAM (see above), Hirshfeld refinement was performed using olex2.refine with NoSpherA2 integrated in the Olex2 interface. Five DFT functionals (BP86, B3LYP, PBE, PBE0, and M06-2X) were tested while all other parameters were kept constant. Each calculation used Orca 6.1 software with the def2-TZVPP basis set and *S* = 1/2, and was refined iteratively until convergence. The Co–H distances (in Å) across all five functionals are as follows: 1.62(4) from PBE, 1.63(4) from PBE0, 1.61(5) from M06-2X, 1.61(4) from BP86, and 1.62(4) from B3LYP. The results of each functional and a representative model are summarized in Figure 5a and Table 1 ().

**Figure 5:**
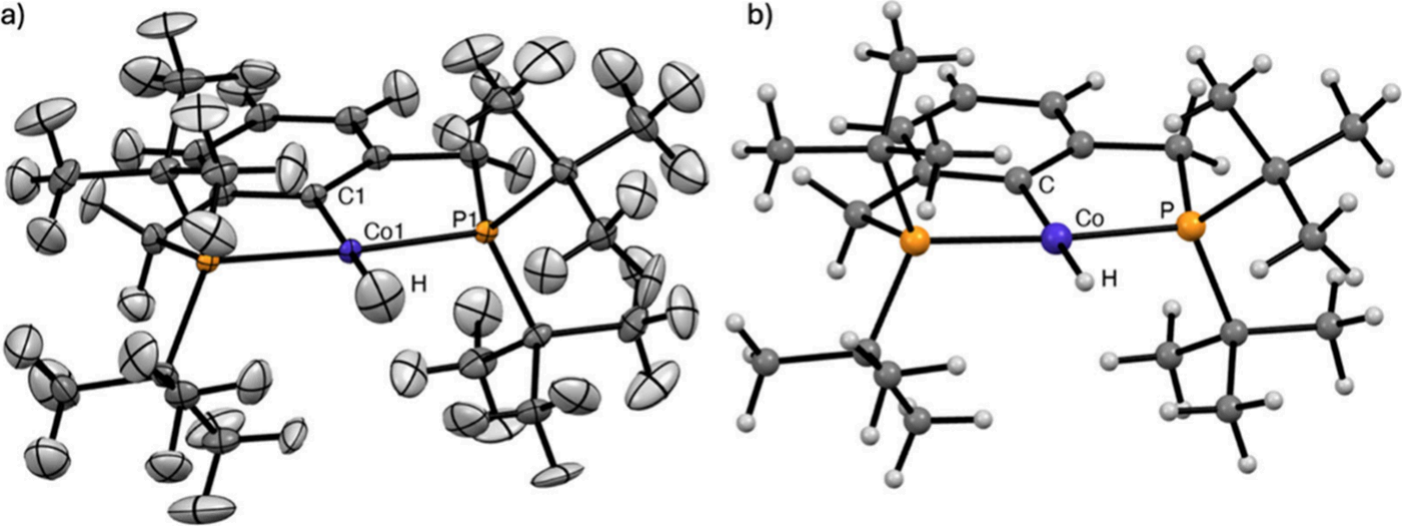
a) ORTEP plot of the X-ray diffraction structure of **CoH** using Hirshfeld atom refinement with the PBE functional. All atoms are refined with anisotropic thermal parameters. Key atoms are labeled for clarity. Ellipsoids drawn at the 50% probability level. b) DFT geometry optimized structure of **CoH** using the PBE functional.

**Table 1:** Comparing the Results of All Functionals in Both HAR and DFT

Functional	Basis Set	Co–H (Å) (HAR)	Co–H (Å) (DFT)
PBE	def2-TZVPP	1.62(4)	1.56
PBE0	def2-TZVPP	1.63(4)	1.56
M06-2X	def2-TZVPP	1.61(5)	1.60
BP86	def2-TZVPP	1.61(4)	1.56
B3LYP	def2-TZVPP	1.62(4)	1.56

### Density Functional Theory (DFT) Calculations

Five DFT functionals were employed for geometry optimization for comparison with subsequent Hirshfeld atom refinement (HAR). The functionals BP86,^58^ B3LYP,^59^ PBE,^60^ PBE0,^61^ and M06-2X^62^ were chosen as representatives of GGA, hybrid, and meta-GGA types, and all models used the def2-TZVPP basis set with an *S* = 1/2 spin state and no solvent correction. The calculations with BP86, B3LYP, PBE, and PBE0 all converged to Co–H distances of 1.56 Å while the M06-2X computation furnished a value of 1.60 Å. The results of the DFT optimizations and a representative model are summarized in Figure 5b and Table 1 ().

### Electron Diffraction (3D-ED/MicroED)

3D-ED/MicroED analysis was performed on ground microcrystallites of **CoH** using a Rigaku Synergy-ED electron diffractometer with a 200 keV (λ = 0.0251 Å) electron beam. Nineteen data sets with at least 60% completeness and 1.50 Å resolution were evaluated (). One data set was chosen that had 89.8% completeness and 0.91 Å resolution after collecting 65° with 2.5 s/deg stage rotation at 96 K. Initial structure solution and refinement used SHELX in Olex2 to build a kinematical model with full anisotropic refinement of all non-hydrogen atoms (Figure 6a). Hydrogen atoms were included in the model at geometrically calculated positions and refined using a riding model (as in IAM section above). The hydride was not yet observed at this stage (Figure 6b). The kinematical model was then imported into Jana2020 for subsequent dynamical refinement.^63^ After initial dynamical refinement, the difference map showed residual electrostatic potential 1.44 Å from the cobalt center. Placing the hydride in this location and further refinement furnished a Co–H distance of 1.51(4) Å (Figure 6c).

**Figure 6:**
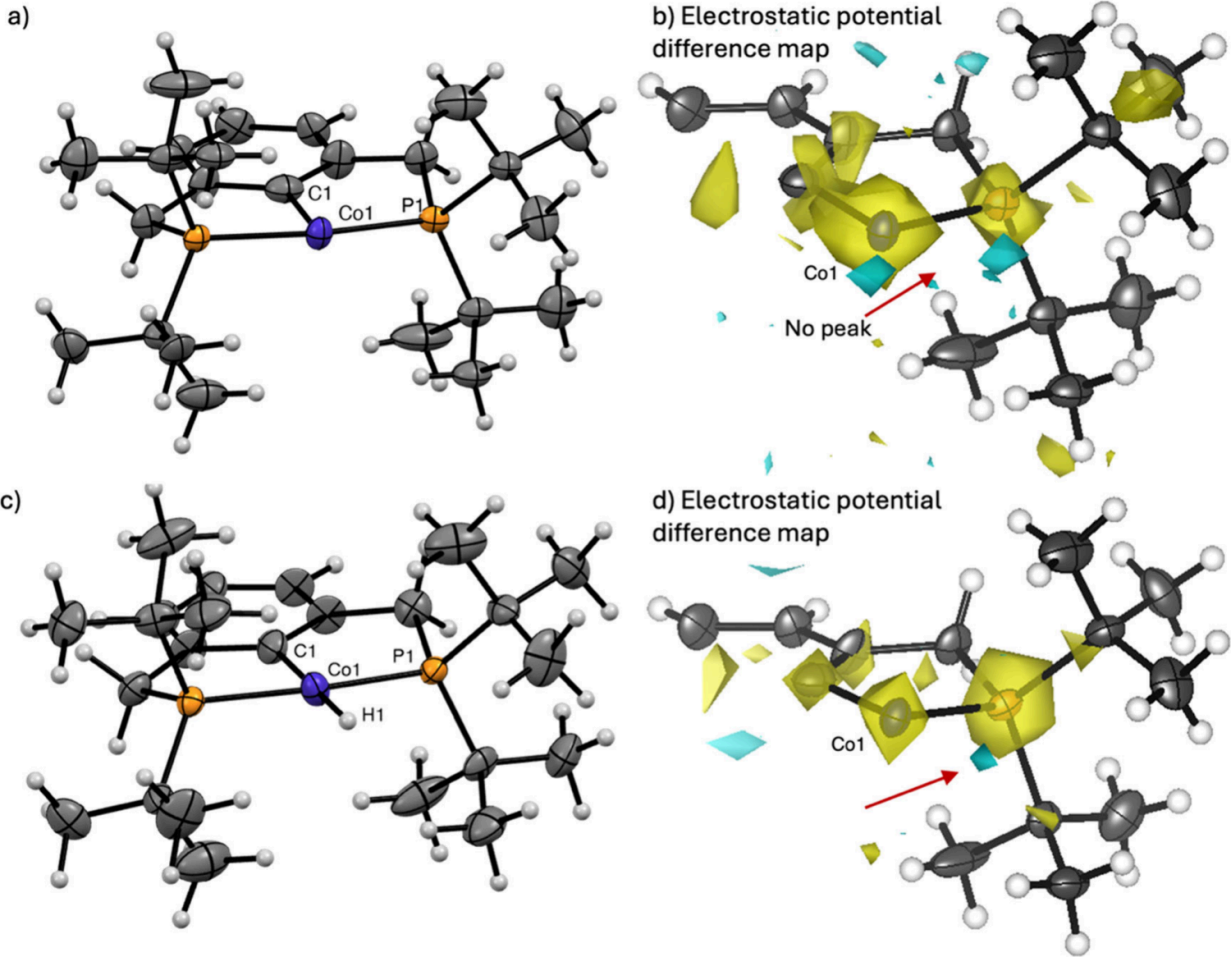
a) ORTEP plot of the electron diffraction structure of **CoH** using the kinematical model. All non-hydrogen atoms are refined with anisotropic thermal parameters. Hydrogens, except for the hydride, use a riding model. Key atoms are labeled for clarity. Ellipsoids drawn at the 50% probability level. b) Difference potential map of the kinematical ED model (yellow indicates positive potential while blue indicates negative). The arrow indicates where potential corresponding to the hydride would be expected. The large potential in plane with Co1 is too far away (>2.5 Å) to be reasonably assigned as the hydride (see for cross-section). c) ORTEP plot of the electron diffraction structure of **CoH** using the dynamical model. All non-hydrogen atoms are refined with anisotropic thermal parameters. Carbon-bound hydrogens use a riding model. Key atoms are labeled for clarity. Ellipsoids drawn at the 50% probability level. d) Difference potential map of the dynamical ED model. The arrow indicates potential corresponding to the hydride in plane with Co1 (see for cross-section).

## Discussion

Neutron diffraction provides the most accurate method for assessing the positions of hydrogen nuclei because they interact strongly with incident neutrons. We therefore use the neutron Co–H distance of 1.54(2) Å as the reference value for this study and compare to the other methods in Table 2. The table gives the standard uncertainties (esd), which is not surprisingly lowest for the neutron diffraction and highest for the traditional IAM refinement of XRD data. We also show the normalized difference, where values of Δ ≲ 1 indicate agreement within combined uncertainty. Figure 7 shows a comparison of all four crystallographically determined structures.

**Table 2:** Refined Co–H Distances from Each Method, Estimated Standard Deviation (esd) Values, and Normalized Differences of Each Crystallographic Refinement^a^

Method	Co–H distance (Å)	esd	Δ
**Neutron diffraction**	**1.54**	**0.02**	**0**
IAM	1.49	0.07	0.69
PBE (HAR)	1.62	0.04	1.79
PBE0 (HAR)	1.63	0.04	2.01
M06-2X (HAR)	1.61	0.05	1.30
BP86 (HAR)	1.61	0.04	1.57
B3LYP (HAR)	1.62	0.04	1.79
**3D-ED/MicroED**	**1.51**	**0.04**	**0.67**

a The normalized distance was calculated using Δ = $|d_{\mathrm{method}}-d_{\mathrm{neutron}}|/$ ​ $\sqrt{{\sigma_{\mathrm{method}}}^{2}+{\sigma_{\mathrm{neutron}}}^{2}}$ , where the denominator is the combined standard uncertainty.

**Figure 7:**
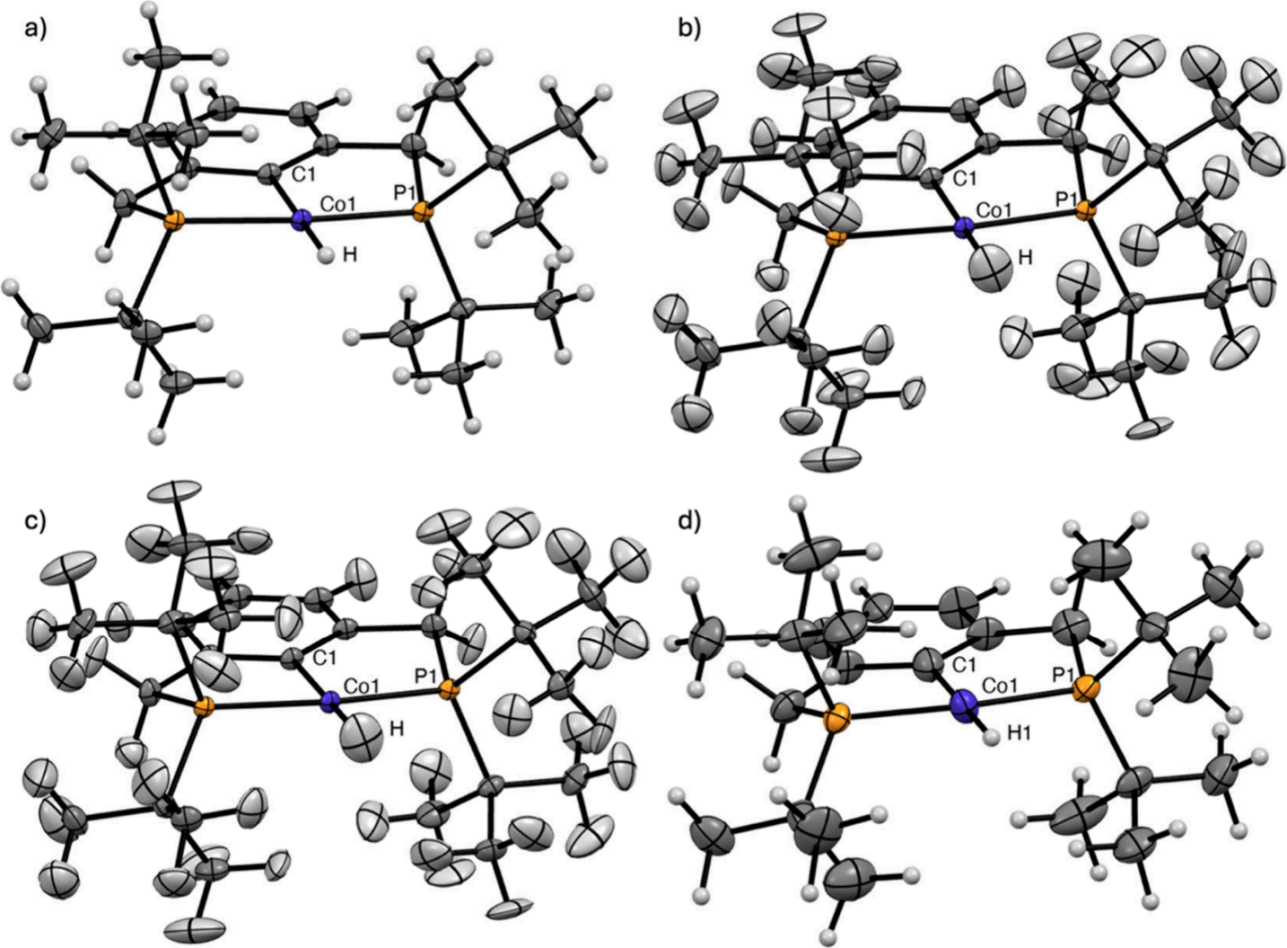
Thermal-ellipsoid plots with 50% ellipsoids indicating anisotropic thermal parameters and small spheres indicating atoms with isotropic thermal parameters. a) X-ray diffraction structure of **CoH** with IAM. b) X-ray diffraction structure of **CoH** with HAR. c) Neutron diffraction structure of **CoH**. d) Dynamically refined electron diffraction structure of **CoH**.

We first analyze the X-ray diffraction (XRD) data. Standard refinement of the XRD data using the IAM yields a Co–H distance of 1.49(7) Å. Though this value is consistent with the neutron benchmark, it has a relatively large esd. As mentioned above, the IAM treats electron density as perfectly spherical and ignores deformations due to bonding or lone pairs.^13^ For hydrogen atoms, this limitation is amplified because X-rays do not interact with the hydrogen nucleus and hydrogen’s one electron is shifted toward the atom to which it is bonded.^7^ A modest shortening bias is generally expected for X–H distances refined from X-ray data under IAM assumptions,^64^ but the most important observation is the larger uncertainty.

To model the aspherical atomic form factors in **CoH**, we turned to Hirshfeld atom refinement as integrated in Olex2 through NoSpherA2.^23^ After identifying the ground spin state in **CoH** as a doublet (*S* = 1/2), we evaluated five common density functionals for transition metals for the wavefunction calculation that led to aspherical form factors (see above). Refinement against these aspherical form factors led to improved figures of merit and C–H bond lengths that are in close agreement with the neutron data across all five functionals (). In contrast, all the Co–H bond lengths from HAR are longer than the neutron reference by 0.07–0.09 Å (Table 1), indicating a reproducible large deviation under the present HAR protocol. We note that using a three-molecule cluster to simulate the local crystal environment () or including relativistic effects in the DFT calculations did not improve the Co–H distances in our refinements.

In 2023, Woźniak and co-workers published a comprehensive study comparing multiple permutations of HAR parameters to IAM and neutron data for ten TM–H compounds.^65^ In their study, HAR reproduced TM–H distances for five cases, while the remaining cases showed either elongation or shortening relative to neutron data. The authors observed improved agreement with higher data quality. In the present system, the X-ray data are high resolution with a favorable data-to-parameter ratio and excellent refinement statistics, which suggests that the persistent Co–H deviation is not readily explained by data quality alone. Instead, the outcome points to protocol sensitivities that may be amplified for open-shell TM–H bonds: specific sources of error could be the density-partitioning scheme used to generate atomic form factors, or correlation between hydride position and displacement parameters. We considered that the crystal environment in the wavefunction could be a source of error, but inclusion of three adjacent molecules in the calculation did not alter the deviation as noted above.

Recent studies from Woźniak also indicate that HAR can improve thermal parameters of TM–H more reliably than the bond lengths themselves.^66^ In an effort to improve the accuracy of Hirshfeld refinement, an alternative partition function has been developed.^67^ While the new partition was only tested on a set of small organic molecules, the method led to improved hydrogen atom parameters in the majority of models. Extending such developments to open-shell transition-metal hydrides in the future is a potential pathway for reducing the systematic bias observed here.

For completeness, we also wanted to compare to bond lengths from pure computations with no crystallographic input. Using the same five DFT functionals employed in our crystallographic refinements, we optimized geometries. Four functionals (BP86, B3LYP, PBE, and PBE0) gave Co–H bond lengths of 1.56 Å, which are in good agreement with the neutron data. However, the Minnesota functional M06-2X converged to a longer value of 1.60 Å. Further examination of the M06-2X optimized structure reveals that the Co–C_aryl_ and Co–P bond lengths are longer, and there is a larger torsion angle in the pincer backbone, than the structures optimized with other functionals. The deviation from the other four functionals could be due to M06-2X being parametrized for main group compounds; this functional was shown to poorly describe 3d TM–H bond strengths.^68^ We note that the HAR Co–H distances are longer than the corresponding DFT equilibrium values for most functionals, which suggests that the systematic error in the HAR quantum refinement arises in the refinement protocol rather than in the underlying DFT description.

Finally, we address the use of 3D-ED/MicroED. When the 3D-ED/MicroED data for **CoH** are refined with a *kinematical* approach, the result is a seemingly three-coordinate cobalt center, (Figure 6a). Further examination of the Fourier difference map does not indicate the presence of the hydride that would complete the coordination sphere. The inability to observe the hydride at this stage is most likely due to dynamical scattering of reflections that are most sensitive to the hydride. Indeed, previous studies have shown that localization of hydrogen atoms with electron diffraction typically requires modeling of dynamical effects.^39,42,44,47^ Once the intensities are modeled with *dynamical* refinement, residual electrostatic potential consistent with the hydride site can be observed in the difference map. Importantly, the refined Co–H distance of 1.51(4) Å is consistent with the neutron distance of 1.54(2) Å with a relatively low uncertainty (Table 2). It is of note that this structure comes from one diffraction data set, which was chosen for its combination of high completeness (89.8%) and resolution (0.91 Å). Another data set that was more complete (99.1%) but had lower resolution (1.03 Å) did not result in localization of the hydride even after dynamical refinement, suggesting that resolution rather than completeness is more important for hydride localizations in the data sets examined here (). Merging these two data sets during refinement improved the thermal model for some non-hydrogen atoms but did not affect the refined Co–H bond length. To further test the resolution dependence on hydride localization, a third data set with 73.5% completeness and 0.98 Å resolution was refined. After initial dynamical refinement, residual electrostatic potential can be observed roughly 1.6 Å from the cobalt center (). However, the model does not converge to a stable solution even with isotropic thermal parameters on every atom, likely due to the low completeness. These observations indicate that hydride localization can be resolution-limited and suggest that it is advisable to report the variation between data sets in future 3D-ED/MicroED hydride studies.

## Conclusion

Here, we describe a comprehensive crystallographic analysis of the Co–H bond length in a cobalt­(II)–hydride complex. Neutron diffraction provides a Co–H distance of 1.54(2) Å, which is used as the reference value for subsequent comparison. Refinement of XRD data with the IAM gives a relatively large uncertainty. When the X-ray data are modeled using aspherical Hirshfeld atom refinement, the C–H distances are improved, but the Co–H distance remains systematically longer than the neutron benchmark under the present protocol. Thus, while coupling crystallography with DFT calculations improves bond length accuracy for X–H bonds to light atoms, the transition metal hydride is not reproduced as accurately relative to the neutron reference using any XRD method. We note as an aside that DFT geometry optimization alone agrees well with the Co–H neutron distance using four of the five functionals tested.

Electron diffraction has an improved ability to scatter from light atoms because the electrostatic potential of both the nucleus and electron density of an atom diffract the incident beam. We show that, for this compound, the hydride can be accurately localized with electron diffraction when coupled with dynamical refinement, giving a Co–H distance of 1.51(4) Å that is consistent with the neutron value and has a relatively low uncertainty. The ability to measure a metal–hydride distance to this degree of accuracy without neutron diffraction demonstrates the effectiveness of 3D-ED/MicroED, as long as the resolution and completeness are sufficiently high. Furthermore, since it is already possible to adapt transmission electron microscopes (TEMs) for electron crystallography, we suggest that this protocol can be widely used. Thus, with the continued development of refinement theory and programs, electron crystallography is poised to be a powerful tool in the chemical crystallographer’s arsenal of methods to determine metal–hydride distances with high reliability.

## Supplementary Material





## Data Availability

The data supporting this article have been included as part of the Supporting Information. Crystallographic data have been deposited at the CCDC with deposition numbers 2542800, 2542969–2542972, and 2542979–2542981.

## Notes

Synthetic procedures, spectroscopy, crystallographic details (PDF)
Crystallographic data files (ZIP)

## References

[1] Organotransition Metal Chemistry: From Bonding to Catalysis; Hartwig, J. F. , Ed.; University Science Books, 2009.

[2] Crabtree, R. The Organometallic Chemistry of the Transition Metals, 8th ed.; Wiley, 2026.

[3] Ji P., Park J., Gu Y., Clark D. S., Hartwig J. F. (2021). Abiotic
Reduction of Ketones with Silanes Catalysed by Carbonic Anhydrase
through an Enzymatic Zinc Hydride. Nat. Chem..

[4] Babón J. C., Esteruelas M. A., López A. M. (2022). Homogeneous Catalysis with Polyhydride
Complexes. Chem. Soc. Rev..

[5] Fishkin A., Morris R. H. (2026). Paramagnetic Transition
Metal Hydride Complexes. Chem. Rev..

[6] Cromer D. T., Mann J. B. (1968). X-Ray Scattering Factors Computed from Numerical Hartree–Fock
Wave Functions. Acta Cryst. A.

[7] Stewart R. F., Davidson E. R., Simpson W. T. (1965). Coherent
X-Ray Scattering for the
Hydrogen Atom in the Hydrogen Molecule. J. Chem.
Phys..

[8] Brown P. J., Fox A. G., Maslen E. N., O’Keefe M. A., Willis B. T. M. (2006). Intensity of Diffracted Intensities. IT C.

[9] Peng L.-M. (1997). Anisotropic
Thermal Vibrations and Dynamical Electron Diffraction by Crystals. Acta Cryst. A.

[10] Rez D., Rez P., Grant I. (1994). Dirac–Fock
Calculations of X-Ray Scattering
Factors and Contributions to the Mean Inner Potential for Electron
Scattering. Acta Cryst. A.

[11] Peng L.-M. (1998). Electron
Scattering Factors of Ions and Their Parameterization. Acta Cryst. A.

[12] Kulik M., Dominiak P. M. (2022). Electron Density
Is Not Spherical: The Many Applications
of the Transferable Aspherical Atom Model. Comput.
Struct. Biotechnol. J..

[13] Koritsanszky T. S., Coppens P. (2001). Chemical Applications of X-Ray Charge-Density
Analysis. Chem. Rev..

[14] Stewart R. F. (1976). Electron
Population Analysis with Rigid Pseudoatoms. Acta Cryst. A.

[15] Hansen N. K., Coppens P. (1978). Testing Aspherical Atom Refinements
on Small-Molecule
Data Sets. Acta Cryst. A.

[16] Dittrich B., Hübschle C. B., Pröpper K., Dietrich F., Stolper T., Holstein J. (2013). The Generalized
Invariom Database (GID). Acta Cryst. B.

[17] Jha K. K., Gruza B., Sypko A., Kumar P., Chodkiewicz M. L., Dominiak P. M. (2022). Multipolar Atom Types from Theory and Statistical Clustering
(MATTS) Data Bank: Restructurization and Extension of UBDB. J. Chem. Inf. Model..

[18] Jha K. K., Gruza B., Kumar P., Chodkiewicz M. L., Dominiak P. M. (2020). TAAM: A Reliable and User Friendly
Tool for Hydrogen-Atom
Location Using Routine X-Ray Diffraction Data. Acta Cryst. B.

[19] Brock C. P., Dunitz J. D., Hirshfeld F. L. (1991). Transferability
of Deformation Densities
among Related Molecules: Atomic Multipole Parameters from Perylene
for Improved Estimation of Molecular Vibrations in Naphthalene and
Anthracene. Acta Cryst. B.

[20] Jelsch C., Pichon-Pesme V., Lecomte C., Aubry A. (1998). Transferability of
Multipole Charge-Density Parameters: Application to Very High Resolution
Oligopeptide and Protein Structures. Acta Cryst.
D.

[21] Hirshfeld F. L. (1977). Bonded-Atom
Fragments for Describing Molecular Charge Densities. Theoret. Chim. Acta.

[22] Capelli S. C., Bürgi H.-B., Dittrich B., Grabowsky S., Jayatilaka D. (2014). Hirshfeld
Atom Refinement. IUCrJ..

[23] Kleemiss F., Dolomanov O. V., Bodensteiner M., Peyerimhoff N., Midgley L., Bourhis L. J., Genoni A., Malaspina L. A., Jayatilaka D., Spencer J. L., White F., Grundkötter-Stock B., Steinhauer S., Lentz D., Puschmann H., Grabowsky S. (2021). Accurate Crystal Structures and Chemical Properties
from NoSpherA2. Chem. Sci..

[24] Jha K. K., Kleemiss F., Chodkiewicz M. L., Dominiak P. M. (2023). Aspherical Atom
Refinements on X-Ray Data of Diverse Structures Including Disordered
and Covalent Organic Framework Systems: A Time–Accuracy Trade-Off. J. Appl. Crystallogr..

[25] Sears V. F. (1992). Neutron
Scattering Lengths and Cross Sections. Neutron
News.

[26] Allen F. H., Bruno I. J. (2010). Bond Lengths in Organic and Metal-Organic
Compounds
Revisited: X—H Bond Lengths from Neutron Diffraction Data. Acta Cryst. B.

[27] Coates L., Cao H. B., Chakoumakos B. C., Frontzek M. D., Hoffmann C., Kovalevsky A. Y., Liu Y., Meilleur F., dos Santos A. M., Myles D. A. A., Wang X. P., Ye F. (2018). A Suite-Level Review
of the Neutron Single-Crystal Diffraction Instruments at Oak Ridge
National Laboratory. Rev. Sci. Instrum..

[28] Garçon M., Bakewell C., Sackman G. A., White A. J. P., Cooper R. I., Edwards A. J., Crimmin M. R. (2019). A Hexagonal Planar Transition-Metal
Complex. Nature.

[29] Jones C. G., Martynowycz M. W., Hattne J., Fulton T. J., Stoltz B. M., Rodriguez J. A., Nelson H. M., Gonen T. (2018). The CryoEM Method MicroED
as a Powerful Tool for Small Molecule Structure Determination. ACS Cent. Sci..

[30] Saha A., Nia S. S., Rodríguez J. A. (2022). Electron
Diffraction of 3D Molecular
Crystals. Chem. Rev..

[31] Gruene T., Wennmacher J. T. C., Zaubitzer C., Holstein J. J., Heidler J., Fecteau-Lefebvre A., De Carlo S., Müller E., Goldie K. N., Regeni I., Li T., Santiso-Quinones G., Steinfeld G., Handschin S., van Genderen E., van Bokhoven J. A., Clever G. H., Pantelic R. (2018). Rapid Structure Determination
of Microcrystalline Molecular Compounds Using Electron Diffraction. Angew. Chem., Int. Ed..

[32] Gallagher-Jones M., Glynn C., Boyer D. R., Martynowycz M. W., Hernandez E., Miao J., Zee C.-T., Novikova I. V., Goldschmidt L., McFarlane H. T., Helguera G. F., Evans J. E., Sawaya M. R., Cascio D., Eisenberg D. S., Gonen T., Rodriguez J. A. (2018). Sub-Ångström
Cryo-EM
Structure of a Prion Protofibril Reveals a Polar Clasp. Nat. Struct. Mol. Biol..

[33] Rodriguez J. A., Ivanova M. I., Sawaya M. R., Cascio D., Reyes F. E., Shi D., Sangwan S., Guenther E. L., Johnson L. M., Zhang M., Jiang L., Arbing M. A., Nannenga B. L., Hattne J., Whitelegge J., Brewster A. S., Messerschmidt M., Boutet S., Sauter N. K., Gonen T., Eisenberg D. S. (2015). Structure
of the Toxic Core of α-Synuclein from Invisible Crystals. Nature.

[34] Andrusenko I., Hamilton V., Mugnaioli E., Lanza A., Hall C., Potticary J., Hall S. R., Gemmi M. (2019). The Crystal Structure
of Orthocetamol Solved by 3D Electron Diffraction. Angew. Chem., Int. Ed..

[35] Yonekura K., Maki-Yonekura S., Takaba K. (2023). Applications and Limitations of Electron
3D Crystallography. Structure.

[36] Kuwabara S. (1959). Accurate Determination
of Hydrogen Positions in NH4Cl by Electron Diffraction. J. Phys. Soc. Jpn..

[37] Honjo G., Shimaoka K. (1957). Determination of Hydrogen
Position in Cubic Ice by
Electron Diffraction. Acta Crystallogr..

[38] Cowley J. M. (1953). Structure
Analysis of Single Crystals by Electron Diffraction. II. Disordered
Boric Acid Structure. Acta Crystallogr..

[39] Palatinus L., Brázda P., Boullay P., Perez O., Klementová M., Petit S., Eigner V., Zaarour M., Mintova S. (2017). Hydrogen Positions
in Single Nanocrystals Revealed by Electron Diffraction. Science.

[40] Goodman P., Lehmpfuhl G. (1968). Observation
of the Breakdown of Friedel’s Law
in Electron Diffraction and Symmetry Determination from Zero-Layer
Interactions. Acta Cryst. A.

[41] Dudka A. P., Avilov A. S., Lepeshov G. G. (2008). Crystal
Structure Refinement from
Electron Diffraction Data. Crystallogr. Rep..

[42] Klar P. B., Krysiak Y., Xu H., Steciuk G., Cho J., Zou X., Palatinus L. (2023). Accurate Structure Models and Absolute Configuration
Determination Using Dynamical Effects in Continuous-Rotation 3D Electron
Diffraction Data. Nat. Chem..

[43] Palatinus L., Jacob D., Cuvillier P., Klementová M., Sinkler W., Marks L. D. (2013). Structure Refinement
from Precession
Electron Diffraction Data. Acta Cryst. A.

[44] Palatinus L., Petříček V., Corrêa C. A. (2015). Structure
Refinement Using Precession Electron Diffraction Tomography and Dynamical
Diffraction: Theory and Implementation. Acta
Cryst. A.

[45] Gruza B., Chodkiewicz M. L., Krzeszczakowska J., Dominiak P. M. (2020). Refinement of Organic
Crystal Structures with Multipolar Electron Scattering Factors. Acta Cryst. A.

[46] Nakai K., Miki K., Kikuchi T., Yamano M. (2025). Detection of Hydrogen
Atoms Using Only 3D ED/MicroED and Contribution to Structure Determining
Salts or Cocrystals. Cryst. Growth Des..

[47] Clabbers M. T. B., Gruene T., van Genderen E., Abrahams J. P. (2019). Reducing Dynamical
Electron Scattering Reveals Hydrogen Atoms. Acta Cryst. A.

[48] Jones C. G., Asay M., Kim L. J., Kleinsasser J. F., Saha A., Fulton T. J., Berkley K. R., Cascio D., Malyutin A. G., Conley M. P., Stoltz B. M., Lavallo V., Rodríguez J. A., Nelson H. M. (2019). Characterization of Reactive Organometallic
Species via MicroED. ACS Cent. Sci..

[49] Woińska M., Grabowsky S., Dominiak P. M., Woźniak K., Jayatilaka D. (2016). Hydrogen Atoms
Can Be Located Accurately and Precisely
by X-Ray Crystallography. Science Advances.

[50] Olech B., Brázda P., Palatinus L., Dominiak P. M. (2024). Dynamical Refinement
with Multipolar Electron Scattering Factors. IUCrJ..

[51] Chodkiewicz M. L., Olech B., Jha K. K., Dominiak P. M., Woźniak K. (2024). Hirshfeld
Atom Refinement and Dynamical Refinement of Hexagonal Ice Structure
from Electron Diffraction Data. IUCrJ..

[52] Rejnhardt P., Gajda R., Woińska M., Parafiniuk J., Giester G., Miletich R., Wu Y., Poręba T., Mezouar M., Sutuła S., Góral T., Dera P., Woźniak K. (2025). Symmetrization of Strong Hydrogen
Bond under High Pressure in Bihydroxide-Ion-Containing NaCu_2_(SO_4_)_2_·H_3_O_2_ Revealed
by Experimental Charge Density, Single-Crystal Electron Diffraction,
and Neutron Diffraction Studies. J. Am. Chem.
Soc..

[53] Semproni S. P., Milsmann C., Chirik P. J. (2014). Four-Coordinate Cobalt Pincer Complexes:
Electronic Structure Studies and Ligand Modification by Homolytic
and Heterolytic Pathways. J. Am. Chem. Soc..

[54] Sang S., Unruh T., Demeshko S., Domenianni L. I., van Leest N. P., Marquetand P., Schneck F., Würtele C., de Zwart F. J., de Bruin B., González L., Vöhringer P., Schneider S. (2021). Photo-Initiated
Cobalt-Catalyzed
Radical Olefin Hydrogenation. Chem.—Eur.
J..

[55] Guard L. M., Hebden T. J., Linn D. E., Heinekey D. M. (2017). Pincer-Supported
Carbonyl Complexes of Cobalt­(I). Organometallics.

[56] Sheldrick G. M. (2015). Crystal
Structure Refinement with SHELXL. Acta Cryst.
C.

[57] Groom C. R., Bruno I. J., Lightfoot M. P., Ward S. C. (2016). The Cambridge Structural
Database. Acta Cryst. B.

[58] Becke A. D. (1988). Density-Functional
Exchange-Energy Approximation with Correct Asymptotic Behavior. Phys. Rev. A.

[59] Miehlich B., Savin A., Stoll H., Preuss H. (1989). Results Obtained with
the Correlation Energy Density Functionals of Becke and Lee, Yang
and Parr. Chem. Phys. Lett..

[60] Perdew J. P., Burke K., Ernzerhof M. (1996). Generalized
Gradient Approximation
Made Simple. Phys. Rev. Lett..

[61] Adamo C., Barone V. (1999). Toward Reliable Density Functional Methods without
Adjustable Parameters: The PBE0Model. J. Chem.
Phys..

[62] Zhao Y., Truhlar D. G. (2008). The M06 Suite of Density Functionals for Main Group
Thermochemistry, Thermochemical Kinetics, Noncovalent Interactions,
Excited States, and Transition Elements: Two New Functionals and Systematic
Testing of Four M06-Class Functionals and 12 Other Functionals. Theor. Chem. Acc..

[63] Petříček V., Palatinus L., Plášil J., Dušek M. (2023). Jana2020 –
a New Version of the Crystallographic Computing System Jana. Z. für Krist. Cryst. Mater..

[64] Madsen, A. Ø. Modeling and Analysis of Hydrogen Atoms. In Electron Density and Chemical Bonding I: Experimental Charge Density Studies; Stalke, D. , Ed.; Springer Berlin Heidelberg: Berlin, Heidelberg, 2012; pp 21–52. 10.1007/430_2011_70.

[65] Woińska M., Pawlędzio S., Chodkiewicz M. L., Woźniak K. (2023). Hirshfeld
Atom Refinement of Metal–Organic Complexes: Treatment of Hydrogen
Atoms Bonded to Transition Metals. J. Phys.
Chem. A.

[66] Woińska M., Hoser A. A., Chodkiewicz M. L., Woźniak K. (2024). Enhancing
Hydrogen Positions in X-Ray Structures of Transition Metal Hydride
Complexes with Dynamic Quantum Crystallography. IUCrJ..

[67] Chodkiewicz M., Woźniak K. (2025). Towards Improved Accuracy of Hirshfeld
Atom Refinement
with an Alternative Electron Density Partition. IUCrJ..

[68] Goodfellow A. S., Bühl M. (2021). Hydricity of 3d Transition Metal
Complexes from Density
Functional Theory: A Benchmarking Study. Molecules.

