# Supplementary material for: Hydride and Seek: Comparing Crystallographic Hydride Placement Techniques with an Open-Shell Cobalt Complex
Source: ACS Cent Sci. 2026 Jun 23;12(7):972–80. doi: 10.1021/acscentsci.6c00632 (PMC13397283; doi:10.1021/acscentsci.6c00632)
Supplement: Supplementary file 1 [file oc6c00632_si_001.pdf]

Supporting Information for

Hydride and Seek: Comparing Crystallographic Hydride Placement Techniques with an Open-Shell Cobalt Complex

Ryan S. Donnelly[1], Theodore J. Gerard[1], Sebastian M. Krajewski[1], Brandon Q. Mercado[1], Xiaoping Wang[2]*, and Patrick L. Holland[1]*

[1] Department of Chemistry, Yale University, New Haven, CT 06520

[2] Neutron Scattering Division, Oak Ridge National Laboratory, Oak Ridge, TN 37831

Contents

General Considerations & Instrumentation	S2
Synthesis and Characterization of **CoH**	S2
NMR Spectrum	S3
Magnetometry	S3
EPR Spectroscopy	S4
IR Spectrum	S5
UV-Visible Absorption Spectrum	S6
Kinematical vs Dynamical Scattering	S6
X-ray Crystallographic Details	S7
Hirshfeld Atom Refinements	S10
Computational Details	S16
Neutron Crystallographic Details	S19
Electron Crystallographic Details	S21
DFT xyz Coordinates	S25
References	S38

General. All manipulations were performed under an atmosphere of Ar gas in an M. Braun glovebox or on a Schlenk line unless otherwise specified. Unless otherwise noted, all solvents were dried via passage through Q5 columns from Glass Contour Co., and stored over 4 Å molecular sieves prior to use. Deuterated solvents were degassed and dried over calcium hydride before storing over molecular sieves prior to use. All structures were visualized with Mercury,[1] and all difference maps were calculated with VESTA.[2] Difference maps for each technique represent the following: nuclear scattering density (neutron diffraction), electron density (X-ray diffraction), and electrostatic potential (electron diffraction). $KHBEt_3$ (1.0 M in THF, Strem), di-*tert*-butylphosphine (Strem), 2-bromo-1,3-bis(bromomethyl)benzene (TCI), and anhydrous cobalt(II) bromide (Alfa Aesar) were used as received without further purification. WARNING! Secondary alkyl phosphines are pyrophoric and care should be taken in handling and disposal. The ^{tBu}PCP–Br ligand and ^{tBu}PCPCoBr (**CoBr**) were synthesized following reported procedures ($^{tBu}PCP = 2,6\text{-}(^tBu_2PCH_2)_2C_5H_3C^-$).[3]

Instrumentation. NMR spectra were acquired on an Agilent 400 MHz spectrometer. ^{1}H NMR chemical shifts were referenced to residual ^{1}H signals from the deuterated solvent from which the sample was prepared. UV-vis spectra were collected on a Cary 60 spectrophotometer using Schlenk-adapted quartz cuvettes with a 1 mm optical path length. IR spectra of solids were obtained with a KBr pellet using a Shimadzu IRTracer-100 with 2 cm^{-1} resolution. Elemental analyses were obtained from the CENTC Elemental Analysis Facility at the University of Rochester. Microanalysis samples were weighed on a PerkinElmer Model AD-6 Autobalance, analyzed on a PerkinElmer 2400 Series II Analyzer, and handled in a VAC Atmospheres argon glovebox. Magnetic susceptibility data were collected using a Quantum Design Physical Properties Measurement System (PPMS). X-band continuous wave (CW) EPR spectra were recorded on samples in 4 mm quartz tubes, at 10 K on a Bruker ELEXYS E500 spectrometer. EPR spectral simulations were performed using EasySpin[4] software operating in MatLab. DC magnetic susceptibility data were simulated using the program MagProp in DAVE 2.0.9.[6] The computer used for HAR has an AMD 9700X CPU, 32 GB DDR5 RAM, and a 1 TB 9100 Pro SSD. Refinements took 15-20 minutes, and no difficulties were encountered during refinement.

*Synthesis of **CoH***

A 20 mL vial was loaded with **CoBr** (21.1 mg, 0.396 mmol) and THF (10 mL). While stirring, $KHBEt_3$ (40 µL , 1.0 M in THF) was added dropwise over 1 minute. A color change from orange to black/yellow was observed 5 minutes after completing the addition. The reaction was stirred for 1 hour at room temperature and then filtered through Celite. Volatile materials were removed under vacuum, and pentane (3 mL) was added to the vial, yielding a dark solution. The pentane solution was filtered through Celite and concentrated to 1 mL before being placed in a –35 °C freezer overnight. Black crystals (12.1 mg, 67 % yield) suitable for scXRD resulted. **^{1}H NMR**

(400 MHz, 25 °C, THF-d_8): δ –12.2 (2H, *m*-aryl), –3.7 (1H, *p*-aryl), 7.5 (36H, *t*Bu), 35.3 (4H, –CH_2–). **$^{31}P\{^1H\}$ NMR** (162 MHz, 25 °C, THF-*d_8*): no clear resonances observed. **FTIR** (KBr, cm^{-1}): 2937(s), 2894(s), 2861(s), 1967(m), 1909(m), 1881(s), 1708(s), 1471(s), 1461(s), 1364(s), 1181(m), 1017(m), 950(w), 829(s), 597(w), 568(w), 484(m). The spectrum contains peaks characteristic of the decomposition product ^{tBu}PCPCo(N_2).[3] **UV-vis** (pentane, λ_{max}) 429 nm. **Elem. Anal.** Found(calcd) for $CoP_2C_{24}H_{44}$ (%): C, 64.34(63.81), H, 9.95(9.82), N 0.07(0.00). Thus, the elemental analysis data are also consistent with some degradation to ^{tBu}PCPCo(N_2), as proposed from the IR data above.

NMR Spectrum

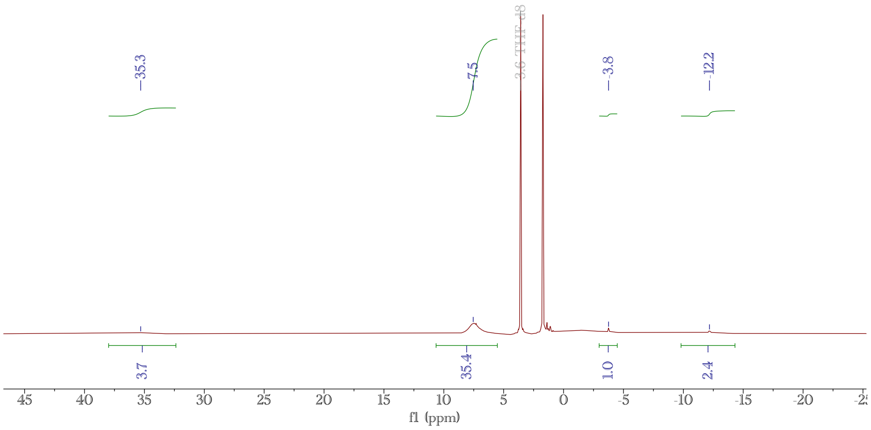

Figure S1. ^{1}H NMR spectrum of ^{tBu}PCPCoH (**CoH**) in THF-d_8.

Magnetometry Data

Magnetic susceptibility data were collected using a Quantum Design Physical Properties Measurement System (PPMS). Magnetic measurements for **CoH** were performed on ground microcrystalline solids. The samples were prepared under an atmosphere of Ar and restrained with eicosane in polyethylene capsules. DC magnetic measurements were collected in the temperature range of 2–300 K. Variable field magnetization curves were collected at 100 K to check for curvature indicative of the presence of ferromagnetic impurities. DC magnetic susceptibility measurements were performed under an applied magnetic field of 0.500 T and corrected for the diamagnetism of the sample and eicosane (-0.0008 emu/mol), estimated using Pascal's constants.[5] A 3% mass correction was applied. DC magnetic susceptibility data were simulated using the program MagProp in DAVE 2.0.9.[6] Magnetic data for **CoH** were modelled according to the spin Hamiltonian $\hat{H} = (g_x+g_y+g_z)\mu BSH$ using $S = 1/2$ and $g_{x,y,z}$ = [4.10 1.88 1.40].

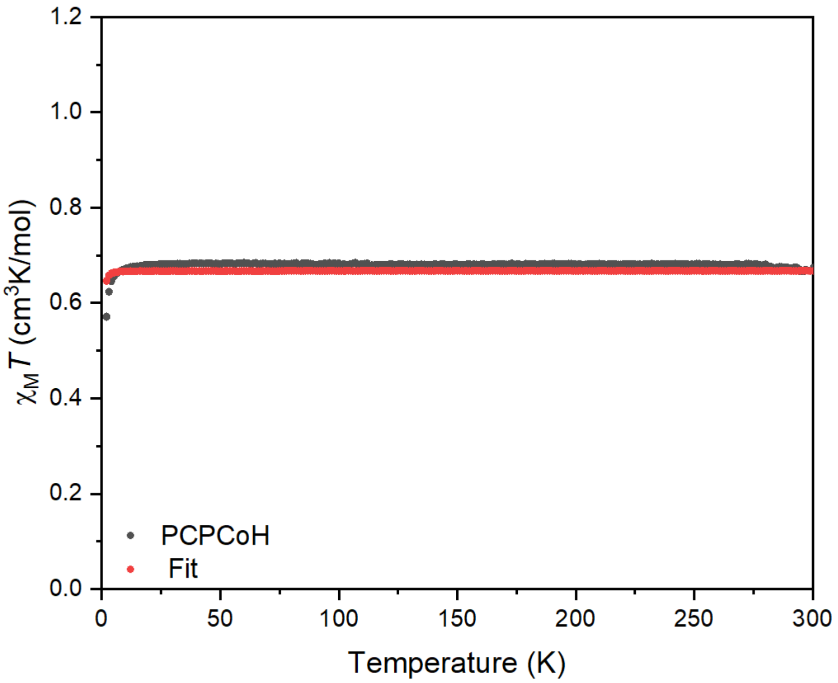

Figure S2. Variable temperature magnetic susceptibility data collected on a polycrystalline sample of **CoH** at 0.500 T. The low temperature $\chi_M T$ value of 0.70 $cm^3 \cdot K/mol$ is consistent with an $S = 1/2$ ground state and is in agreement with its X-band EPR spectrum.

EPR Spectroscopy

Continuous-wave EPR spectra were recorded at X-band frequency (9.352 GHz) in perpendicular mode using a Bruker ELEXSYS EPR Spectrometer equipped with an ER 049X microwave bridge and SHQ resonator. The data were collected using the following parameters: modulation frequency of 100 kHz, modulation amplitude of 5 G, sweep time of 168 s, and microwave power of 0.2 mW for **CoH**. The spectrum was simulated using the pepper function in EasySpin7 with the spin Hamiltonian $\hat{H} = (g_x+g_y+g_z)\mu BSH$ using $S = 1/2$. The spectrum for **CoH** was simulated using a one-component fit.

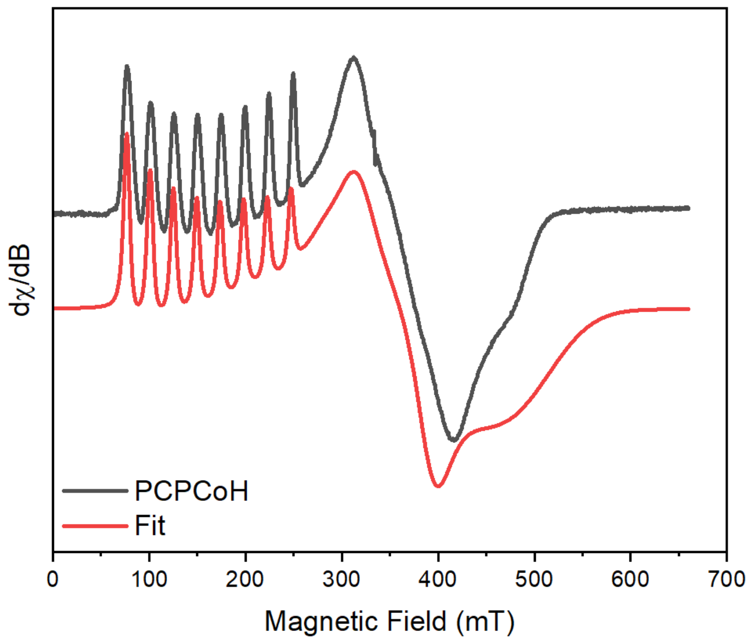

Figure S3. EPR spectrum of ^{tBu}PCPCoH (**CoH**) in toluene. Data were collected at 10 K using a 5 mM solution. The EPR spectrum was simulated as an $S = 1/2$ spin system with $g_{x,y,z} = [4.10, 1.88, 1.40]$ and $A(^{59}Co, I = 7/2) = [1391, 270, 200]$ MHz.

Infrared Absorption Spectrum

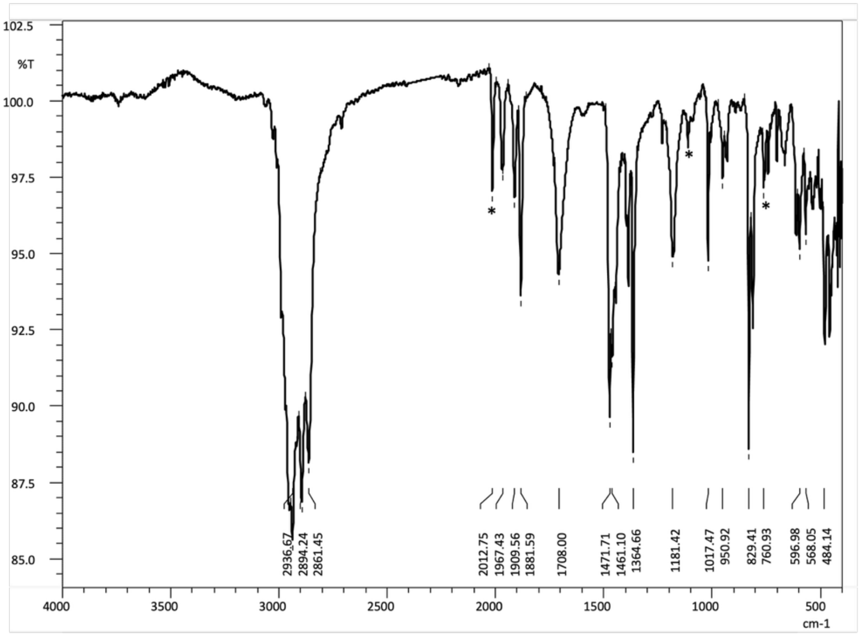

Figure S4. FTIR spectrum of **CoH**. The Co–H band is assigned at 1708 cm^{-1} in agreement with calculated frequencies (1703 cm^{-1} from B3LYP calculation, see below). Bands labeled with asterisks correspond to decomposition products, likely ^{tBu}PCPCo(N_2).

UV-visible Spectrum

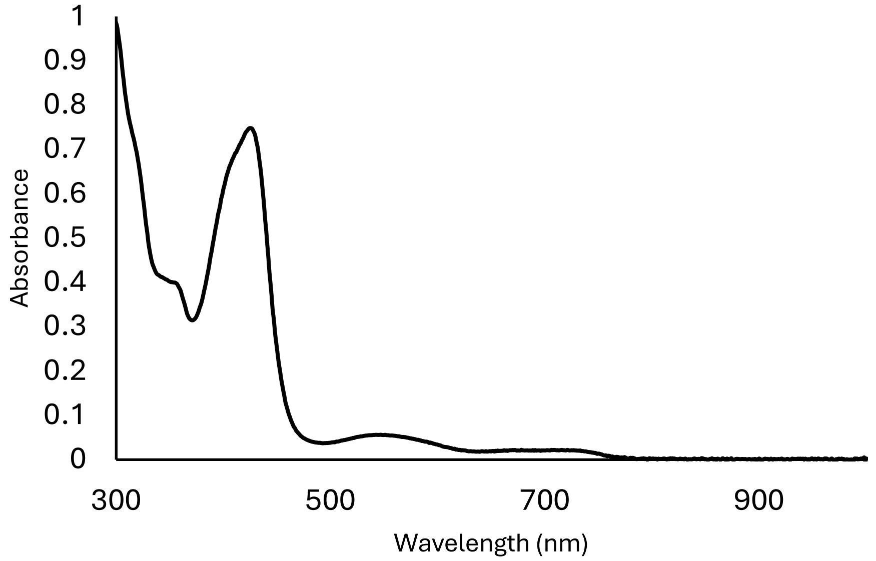

Figure S5. UV-vis spectrum of ^{tBu}PCPCoH (**CoH**) in pentane using a 2.98 mM solution with a 1 mm path length cell.

Kinematical vs. Dynamical Scattering

In the kinematical approximation used for conventional scXRD, each incident photon is assumed to undergo a single scattering event, and the measured reflection intensities are proportional to the squared magnitude of the structure factors $I_{hkl} \propto F_{hkl}F_{hkl}^{*} = |F_{hkl}|^{2}$. Each structure factor comes from the summation over all atoms in the unit cell given by $F(hkl) = \sum_{j=1}^{N} f_j e^{2\pi i(hx_j+ky_j+lz_j)}$, where x_j, y_j, and z_j are the positional coordinates of the jth atom, and f_j is the atomic form factor of the jth atom. The atomic form factors come from the Fourier transform of the electron density of an atom, thereby making f_j directly proportional to atomic Z, meaning that heavier atoms contribute more to observed intensities. Since each photon undergoes a single scattering event, the observed intensities are independent of each other.

However, the kinematical scattering model has limited application to ED since electrons interact more strongly with crystals than X-rays. As a result, one incident electron can undergo multiple scattering events. This is known as dynamical scattering. Because an electron has the potential to scatter multiple times, the relationship $I_{hkl} \propto |F_{hkl}|^{2}$ is no longer valid as intensities for a given set of reflections become dependent on each other. Furthermore, multiple scattering can lead to the observation of reflections that should be symmetry forbidden, hindering subsequent structure solution and refinement. The severity of dynamical scattering effects is directly related to the thickness of a crystal, underlining the need for small samples in 3DED/MicroED analysis.

scXRD

Low-temperature diffraction data (ω-scans) were collected on a Rigaku MicroMax-007HF diffractometer coupled to a Dectris Pilatus3R detector with Mo Kα (λ = 0.71073 Å) for the structure of **CoH**. The diffraction images were processed and scaled using Rigaku Oxford Diffraction software.[7] The structure was solved with SHELXT[8] and was refined against F^2 on all data by full-matrix least squares with SHELXL[9] in Olex2.[10] All non-hydrogen atoms were refined with anisotropic thermal parameters. Hydrogen atoms, except for the hydride, were included in the model at geometrically calculated positions and refined using a riding model. The isotropic displacement parameters of all C–H hydrogen atoms were fixed to 1.2 times the U value of the atoms to which they are linked (1.5 times for methyl groups). The crystal structure of **CoH** reveals a cobalt center ligated by two phosphorus atoms and the central carbon of the pincer ligand. The Co–P bond lengths are 2.1653(2) Å, and the Co–C_{aryl} distance is 1.9627(14) Å. All of these distances are as expected for PC(aryl)P pincers.[11] After adding hydrogen atoms on carbon atoms using calculated positions in a riding model, the hydride can be tentatively identified as the largest difference peak. Placing the hydride and refining with the IAM results in a Co–H distance of 1.49(7) Å.

Table S1. X-ray diffraction data and structure refinement using IAM for **CoH**.

Identification code	007c-25019	
Empirical formula	$C_{24}H_{44}P_2Co$	
Formula weight	453.46	
Temperature	100(3) K	
Wavelength	0.71073 Å	
Crystal system	Tetragonal	
Space group	*I*4₁*cd*	
Unit cell dimensions	a = 16.1164(2) Å	α = 90°
	b = 16.1162(2) Å	β = 90°
	c = 19.1411(3) Å	γ = 90°
Volume	4971.689(15) $Å^3$	
Z	8	
Density (calculated)	1.212 g/cm^3	
Absorption coefficient	0.826 mm^{-1}	
F(000)	1960	
Crystal size	0.4 x 0.4 x 0.5 mm	
Crystal color and habit	black prism	
Diffractometer	Rigaku MicroMax-007HF, Dectris Pilatus3R detector	
Theta range for data collection	2.186, 44.766	
Index ranges	-31<=h<=31, -31<=k<=28, -37<=l<=37	
Reflections collected	61133	
Independent reflections	10142 [R(int) = 4.61 %]	
Observed reflections (I > 2sigma(I))	8513	
Completeness to theta = 50.5°	100 %	
Absorption correction	multi-scan	
Min. and max. transmission	0.666 and 1.000	
Solution method	SHELXT 2018/2 (Sheldrick, 2018)	
Refinement method	SHELXL 2019/3 (Sheldrick, 2015)	
Data / restraints / parameters	8513 / 0 / 133	
Goodness-of-fit on F^2	1.017	
Final R indices [I>2sigma(I)]	R1 = 2.81%, wR2 = 6.68%	
R indices (all data)	R1 = 4.12%, wR2 = 7.11%	
Largest diff. peak and hole	0.5 and –0.4 e.$Å^{-3}$	

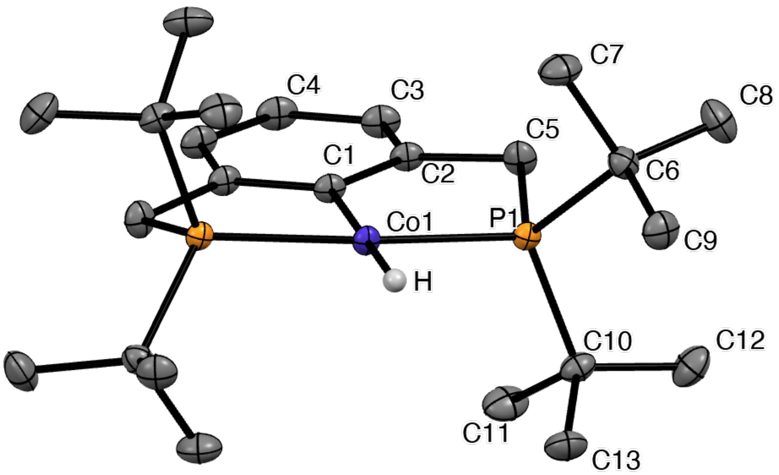

Figure S5. Full numbering scheme of the X-ray crystal structure using the IAM of **CoH** with 50% thermal ellipsoid probability levels. The hydrogen atoms, except the hydride, are omitted for clarity. Complete structural details can be found in the CIF (CCDC number 2542969).

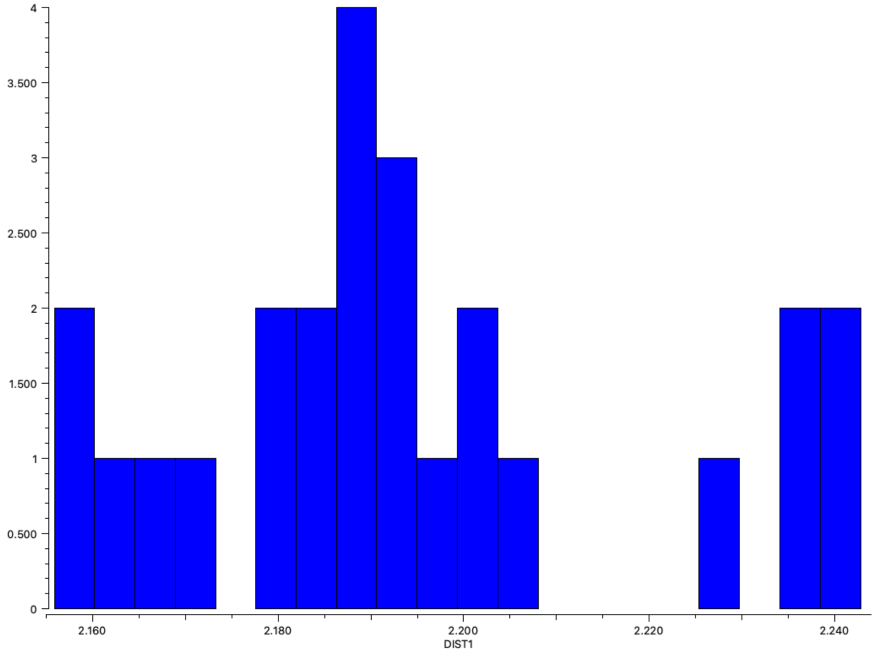

Figure S6. Histogram of Co–P bond lengths as reported in the CCDC for 24 (^{R}PCP)CoL complexes. Average Co–P bond length is 2.20(3) Å.[1]

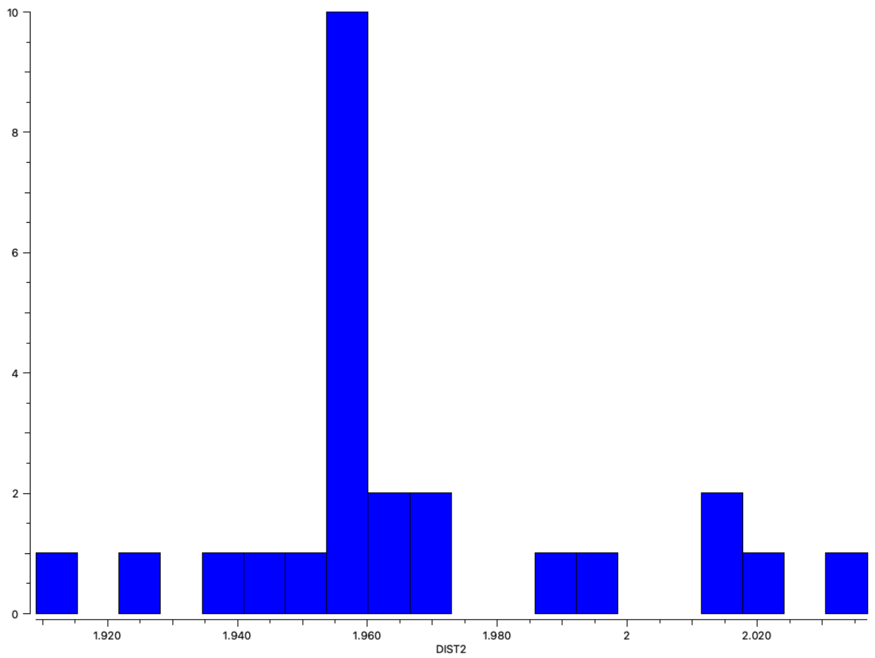

Figure S7. Histogram of Co–C_{aryl} bond lengths as reported in the CCDC for 24 (^{R}PCP)CoL complexes. Average Co–C_{aryl} bond length is 1.97(3) Å.[1]

HAR Data

High-resolution X-ray data were collected on **CoH** to 0.50 Å (λ = 0.7107 Å) as described above. After solving and refining the structure with the IAM (see above), Hirshfeld refinement was performed using olex2.refine with NoSpherA2 integrated in the Olex2 interface.[12] A sample input file is listed below. Five DFT functionals (BP86, B3LYP, PBE, PBE0, and M06-2X) were tested while all other parameters were kept constant. Each refinement used ORCA 6.1 software, the def2-TZVPP basis set, high integration accuracy, TightSCF, Normal Convergence, No AFIX, HAniso, and was refined iteratively until convergence (**Figure S8**). The Co–H distance (in Å) across all five functionals are as follows: 1.62(4) from PBE, 1.63(4) from PBE0, 1.61(5) from M06-2X, 1.61(4) from BP86, and 1.62(4) from B3LYP. The results of each functional and models are summarized in **Figures S9-S13**.

Input file:

! NoPop MiniPrint def2-TZVPP BP DefGrid2 NoFinalGridX TightSCF NormalConv def2/J RIJCOSX

%pal

nprocs 12 end

%maxcore 1280.0

```
%coords
CTyp xyz
charge 0
mult 2
units angs
coords
```

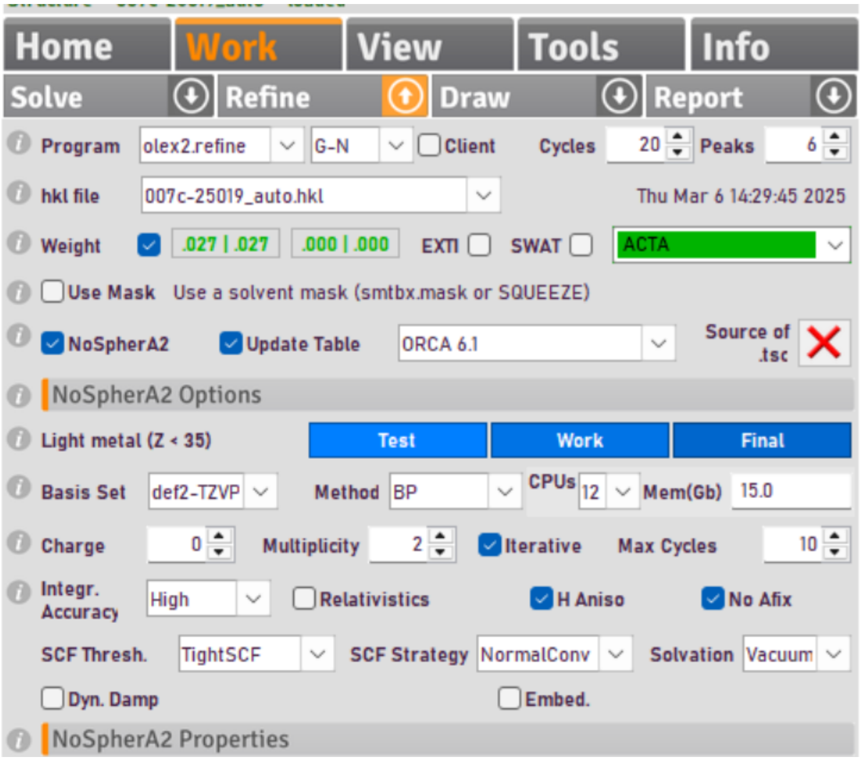

Figure S8. Representative image of the HAR settings used in NoSpherA2.

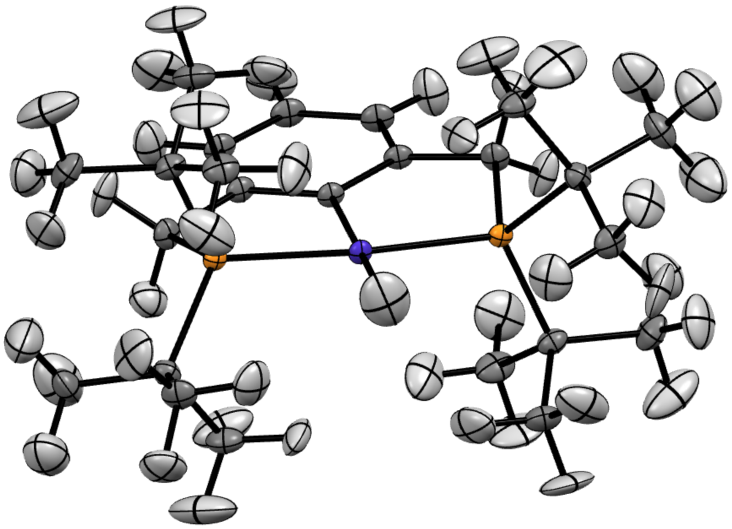

Figure S9. ORTEP plot of the XRD structure of **CoH** using Hirshfeld atom refinement coupled with the PBE DFT functional. Thermal ellipsoids drawn at 50% probability. The Co–H bond length is 1.62(4) Å.

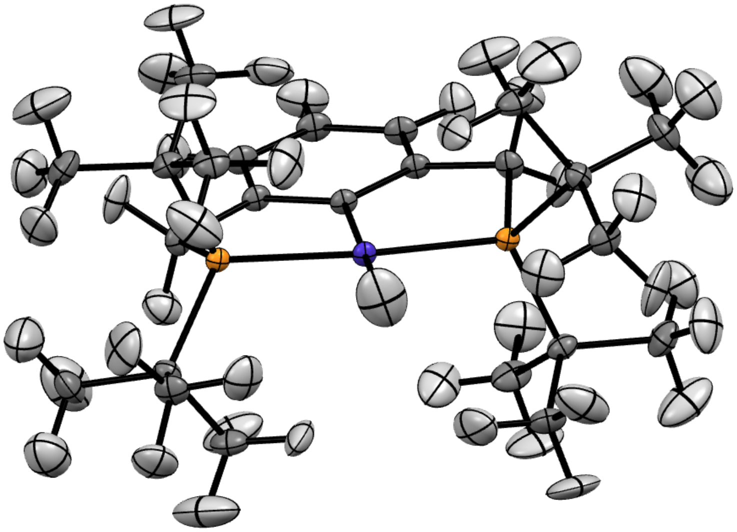

Figure S10. ORTEP plot of the XRD structure of **CoH** using Hirshfeld atom refinement coupled with the PBE0 DFT functional. Thermal ellipsoids drawn at 50% probability. The Co–H bond length is 1.63(4) Å.

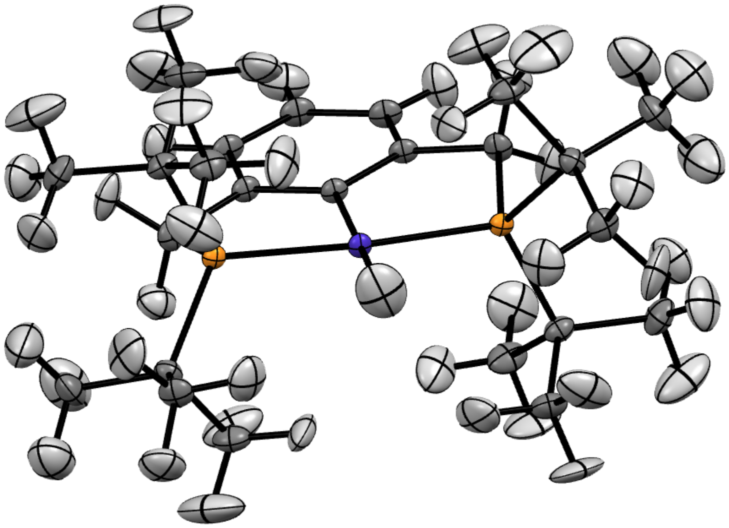

Figure S11. ORTEP plot of the XRD structure of **CoH** using Hirshfeld atom refinement coupled with the BP86 DFT functional. Thermal ellipsoids drawn at 50% probability. The Co–H bond length is 1.61(4) Å.

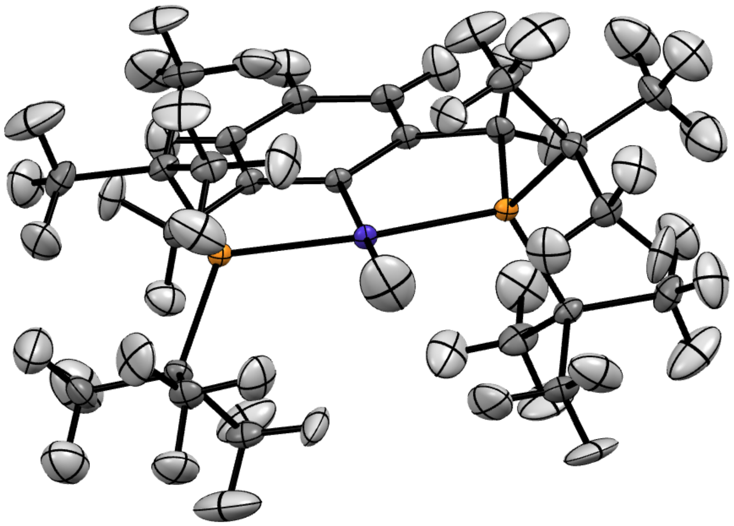

Figure S12. ORTEP plot of the XRD structure of **CoH** using Hirshfeld atom refinement coupled with the B3LYP DFT functional. Thermal ellipsoids drawn at 50% probability. The Co–H bond length is 1.62(4) Å.

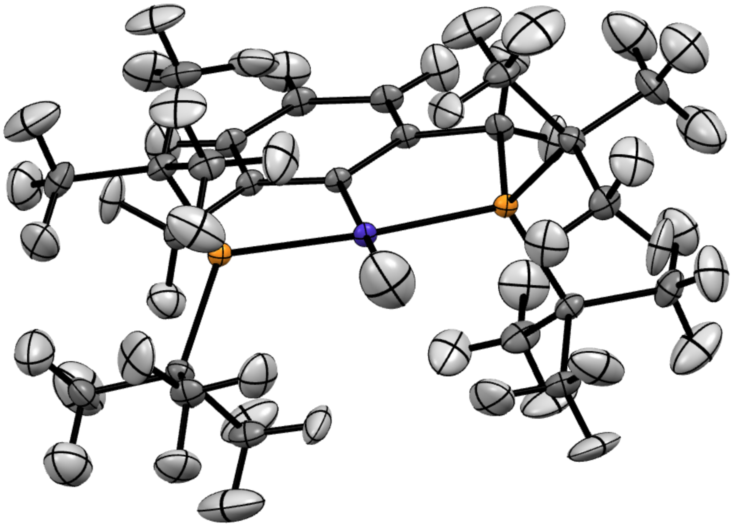

Figure S13. ORTEP plot of the XRD structure of **CoH** using Hirshfeld atom refinement coupled with the M06-2X DFT functional. Thermal ellipsoids drawn at 50% probability. The Co–H bond length is 1.61(5) Å.

Table S2. Comparison of C–H bond lengths (Å) among neutron diffraction and the HAR structures.

Bond	Neutron	PBE	PBE0	BP86	B3LYP	M06-2X
C3–H3	1.075(10)	1.085(12)	1.088(12)	1.083(12)	1.083(12)	1.084(12)
C4–H4	1.067(10)	1.10(3)	1.10(3)	1.10(3)	1.10(3)	1.10(3)
C5–H5A	1.073(13)	1.106(11)	1.111(12)	1.105(11)	1.108(11)	1.109(12)
C5–H5B	1.070(12)	1.093(12)	1.092(12)	1.093(12)	1.093(13)	1.091(13)
C7–H7A	1.104(13)	1.101(14)	1.108(15)	1.101(14)	1.106(15)	1.103(15)
C7–H7B	1.104(13)	1.100(11)	1.104(11)	1.099(11)	1.101(11)	1.099(11)
C7–H7C	1.077(15)	1.088(13)	1.093(14)	1.088(13)	1.093(14)	1.091(14)
C8–H8A	1.103(17)	1.050(14)	1.055(14)	1.049(14)	1.054(14)	1.053(15)
C8–H8B	1.092(13)	1.084(14)	1.080(15)	1.084(14)	1.081(15)	1.077(15)
C8–H8C	1.096(13)	1.082(14)	1.089(14)	1.082(14)	1.085(14)	1.087(14)
C9–H9A	1.102(14)	1.108(12)	1.112(12)	1.107(12)	1.108(12)	1.111(12)
C9–H9B	1.077(12)	1.090(13)	1.092(13)	1.090(13)	1.089(13)	1.087(13)
C9–H9C	1.095(14)	1.101(11)	1.104(11)	1.101(11)	1.104(12)	1.102(12)
C11–H11A	1.082(17)	1.089(14)	1.091(15)	1.089(14)	1.094(15)	1.087(15)
C11–H11B	1.103(13)	1.101(14)	1.103(14)	1.099(14)	1.100(14)	1.102(14)
C11–H11C	1.067(15)	1.076(11)	1.081(11)	1.075(11)	1.077(11)	1.079(12)
C12–H12A	1.100(14)	1.089(14)	1.088(14)	1.088(14)	1.087(14)	1.086(14)
C12–H12B	1.074(15)	1.106(13)	1.108(13)	1.106(13)	1.108(13)	1.109(13)
C12–H12C	1.100(14)	1.083(12)	1.085(12)	1.081(12)	1.079(12)	1.083(12)
C13–H13A	1.072(14)	1.089(14)	1.091(15)	1.090(14)	1.094(14)	1.087(15)
C13–H13B	1.088(13)	1.091(13)	1.093(13)	1.090(13)	1.091(13)	1.090(13)
C13–H13C	1.064(16)	1.109(13)	1.110(13)	1.108(13)	1.109(13)	1.108(13)

Table S3. Comparing selected figures of merit among the 5 HAR structures.

	PBE	PBE0	BP86	B3LYP	M06-2X
Parameters	324	324	324	324	324
Goodness of fit	0.990	0.983	0.990	0.988	0.985
R(%) (reflections)	2.45 (8513)	2.45 (8513)	2.45 (8513)	2.46 (8513)	2.47 (8513)
wR2(%) (reflections)	5.46 (10142)	5.49 (10142)	5.46 (10142)	5.50 (10142)	5.54 (10142)
$\Delta\rho$ min/max	–0.9, 0.5	–0.9, 0.5	–0.8, 0.5	–0.9, 0.5	–0.9, 0.5
Refined H positions	yes				
H thermal motions	anisotropic				

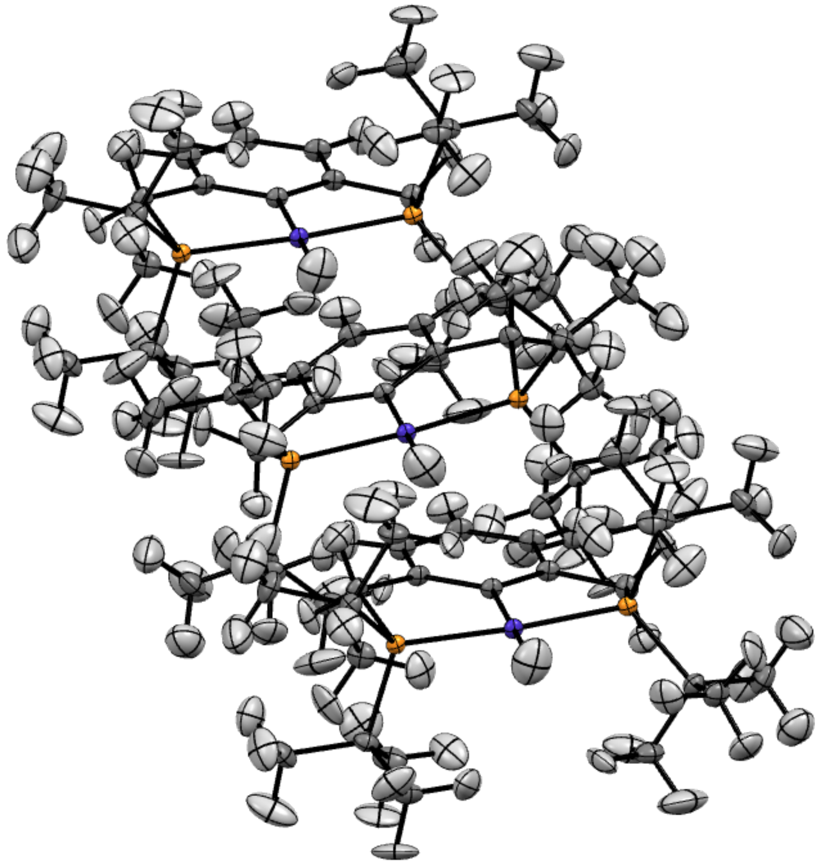

Figure S14. Three molecule cluster used to simulate the crystal environment in HAR calculations at the PBE level of theory. All other NoSpherA2 parameters were kept as described above. Both an antiferromagnetic (overall $S = 1/2$) and a ferromagnetic (overall $S = 3/2$) local coupling regimes were calculated. Both refinements converged to a Co–H distance of 1.61(4) Å.

Computational Details

Five DFT functionals were employed for geometry optimization in ORCA 6.0.1.[13] The functionals BP86, B3LYP, PBE, PBE0, and M06-2X were chosen as representatives of GGA, hybrid, and meta-GGA types, and all models used the def2-TZVPP basis set. The calculations with BP86, B3LYP, PBE, and PBE0 all converged to Co–H distances of 1.56 Å while the M06-2X computation furnished a value of 1.60 Å. The results of the DFT optimizations and models are summarized in **Figures S14-S18.** An example input for geometry optimization calculations on **CoH** can be seen below:

```
! PBE D3BJ def2-TZVPP def2/j UKS
! TightSCF TightOpt NumFreq NormalPrint

%pal nproc 20
end

%scf MaxIter 1800
end
```

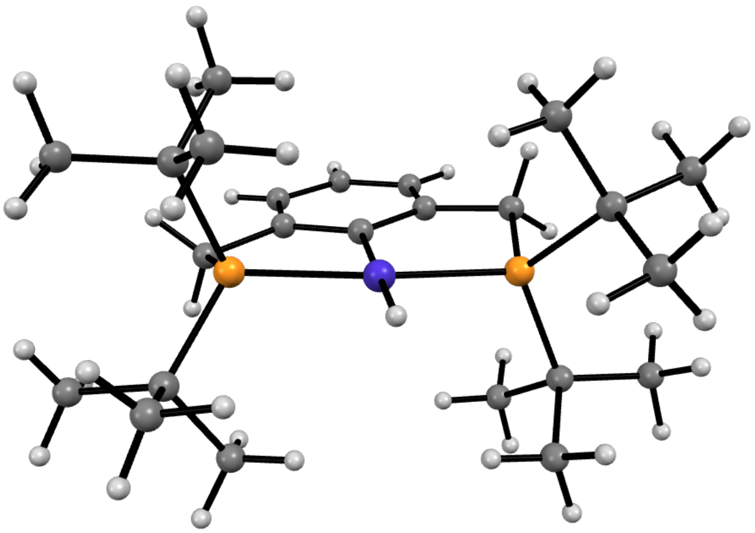

Figure S15. DFT optimized structure of **CoH** with the PBE functional. The Co–H bond length is 1.56 Å.

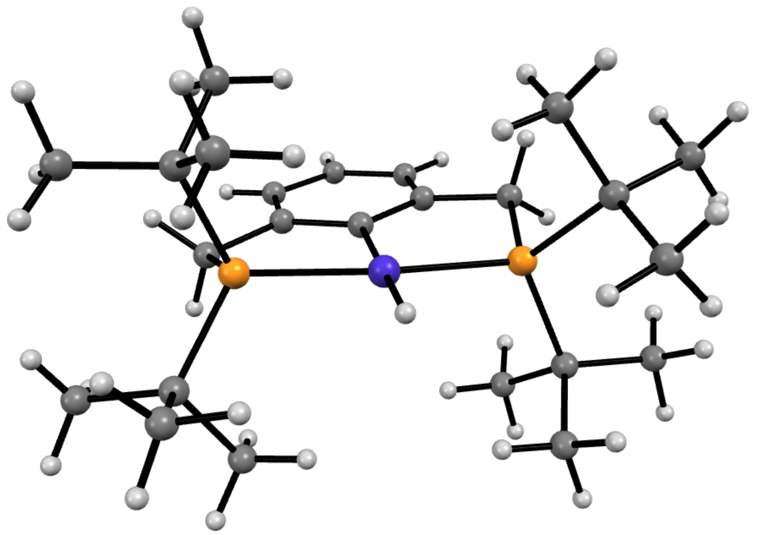

Figure S16. DFT optimized structure of **CoH** with the PBE0 functional. The Co–H bond length is 1.56 Å.

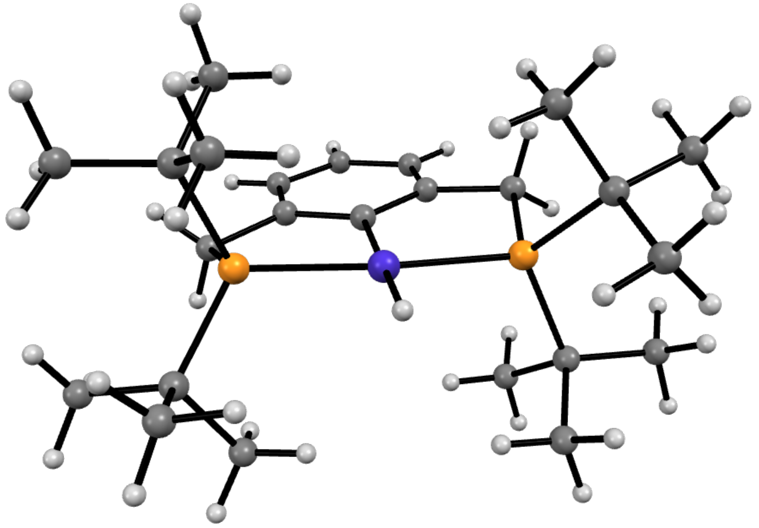

Figure S17. DFT optimized structure of **CoH** with the BP86 functional. The Co–H bond length is 1.56 Å.

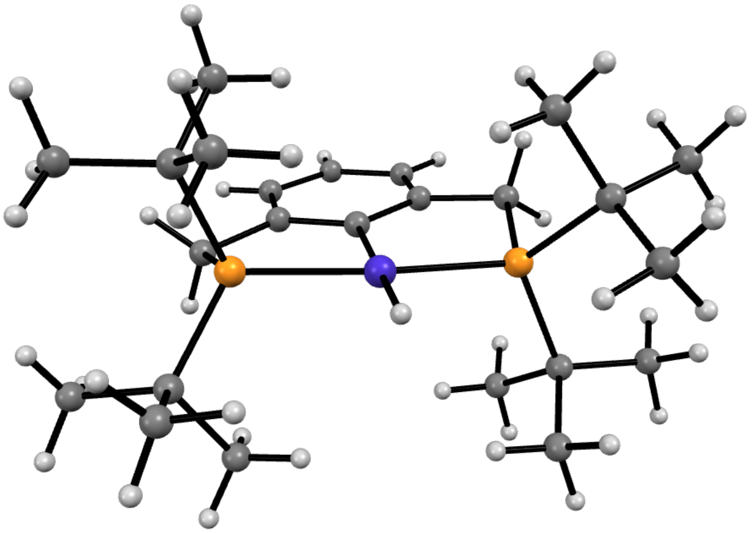

Figure S18. DFT optimized structure of **CoH** with the B3LYP functional. The Co–H bond length is 1.56 Å.

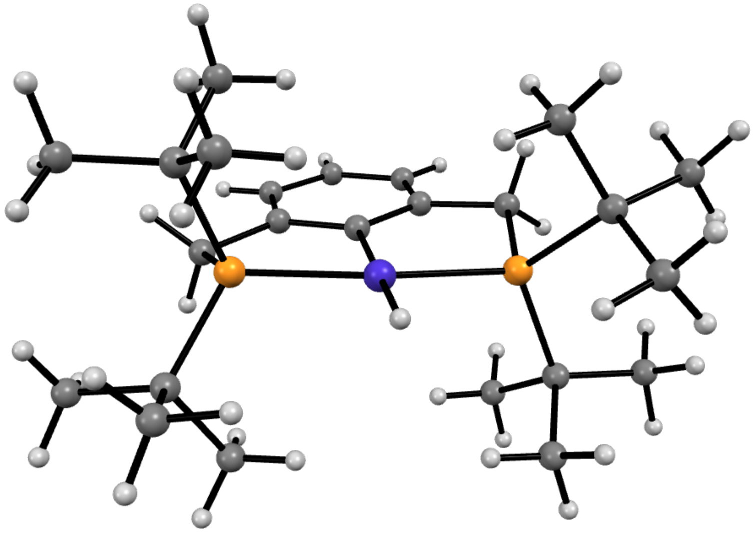

Figure S19. DFT optimized structure of **CoH** with the M06-2X functional. The Co–H bond length is 1.60 Å.

Single Crystal Neutron Diffraction

Single crystal neutron diffraction for **CoH** was carried out on the TOPAZ beamline at the Spallation Neutron Source, Oak Ridge National Laboratory, equipped with a large-area array of Anger cameras that record neutron time-of-flight events in wavelength-resolved Laue mode. TOPAZ uses a large area array of Anger cameras to record neutron time-of-flight events in wavelength-resolved Laue mode. A plate-like single crystal (1.18 × 0.88 × 0.12 mm) was mounted on a MiTeGen loop with cyanoacrylate adhesive and measured at 100 K using a cryogen-free Cobra open-flow cooler (Oxford Cryosystems). The data collection strategy was planned with NeuXtalViz.[14] Peaks were integrated with a multiresolution machine-learning method in three-dimensional HKL space.[15] Data reduction and normalization followed established TOPAZ protocols,[16] including corrections for the Lorentz factor, the time-of-flight spectrum, and detector efficiency. A Gaussian correction was applied for absorption. The crystal shape was determined using the same crystal and face indexing with a Rigaku benchtop Xtallab miniII. The corresponding linear attenuation used for absorption correction was $\mu = 0.16863 + 0.13892\ \lambda\ \mathrm{mm}^{-1}$ with λ in Å. Reduced intensities were exported in SHELX HKLF2 format[9] with the per-reflection neutron wavelength retained. The hydride position was located from the difference map of the neutron scattering density, which is negative for hydrogen, with a height of –6.3(2) fm $Å^{-3}$. All the hydrogen atoms are refined with anisotropic thermal parameters. Structure solution and refinement to convergence were performed in SHELXL.

Table S4. Experimental Details for Neutron Refinement of **CoH**.

Identification code	PCPCoH1	
Empirical formula	$C_{24}H_{44}P_2Co$	
Formula weight	453.46	
Temperature	100(2) K	
Radiation type	Neutrons, λ = 0.58 – 3.50 Å	
Crystal system	Tetragonal	
Space group	$I4_1cd$	
Unit cell dimensions	a = 16.1164(2) Å	α = 90°
	b = 16.1162(2) Å	β = 90°
	c = 19.1411(3) Å	γ = 90.
Volume	4971.689(15) Å^3	
Z	8	
Density (calculated)	1.212g/cm^3	
Absorption coefficient	0.16863 + 0.13892 λ mm^{-1}	
F(000)	1960	
Crystal size	1.18 × 0.88 × 0.12 mm	
Crystal color and habit	purple plate	
Diffractometer	TOPAZ	
Theta range for data collection	7.804, 78.262	
Index ranges	-18<=h<=21, -21<=k<=21, -25<=l<=25	
Reflections collected	6339	
Independent reflections	2317 [R(int) = 10.5 %]	
Observed reflections (I > 2sigma(I))	1884	
Completeness to theta = 29.69°	88.9 %	
Absorption correction	Gaussian	
Min. and max. transmission	0.652 and 0.946	
Solution method	SHELXT 2018/2 (Sheldrick, 2018)	
Refinement method	SHELXL 2019/3 (Sheldrick, 2015)	
Data / restraints / parameters	2317 / 1 / 323	
Goodness-of-fit on F^2	1.151	
Final R indices [I>2sigma(I)]	R1 = 6.03 %, wR2 = 10.7 %	
R indices (all data)	R1 = 8.12 %, wR2 = 12.6 %	
H-atom treatment	All H-atom parameters refined	
Largest diff. peak and hole	0.91 and –0.73 fm Å^{-3}	

Electron Diffraction Details

Samples for electron diffraction were prepared through crushing crystals between two microscope slides then applying to 3 mm copper grids with 200 mesh and lacey carbon support film (TedPella, Inc.). MicroED analysis was conducted with low-temperature diffraction data (ω-scans) collected on a Rigaku Synergy-ED electron diffractometer coupled to a HyPix-ED detector with a 200 keV ($\lambda = 0.0251$ Å) electron beam for the structure of **CoH**. The diffraction images were processed and scaled using Rigaku Oxford Diffraction software. The structure was solved with SHELXT and was refined against F^2 on all data by full-matrix least squares with SHELXL. All non-hydrogen atoms were refined with anisotropic thermal parameters. The atom labeled carbon C3 had its ADPs constrained. Hydrogen atoms, except for the hydride, were included in the model at geometrically calculated positions and refined using a riding model. The isotropic displacement parameters of all hydrogen atoms were fixed to 1.2 times the U value of the atoms to which they are linked. The hydride was not localized with the kinematical model. The kinematic model was then imported into Jana2020 for subsequent dynamical refinement.[17] After initial dynamical refinement, the difference map showed residual electron density 1.44 Å from the cobalt center. Placing the hydride on this residual density and further refinement furnished a Co–H distance of 1.51(4) Å. The structure was refined until convergence.

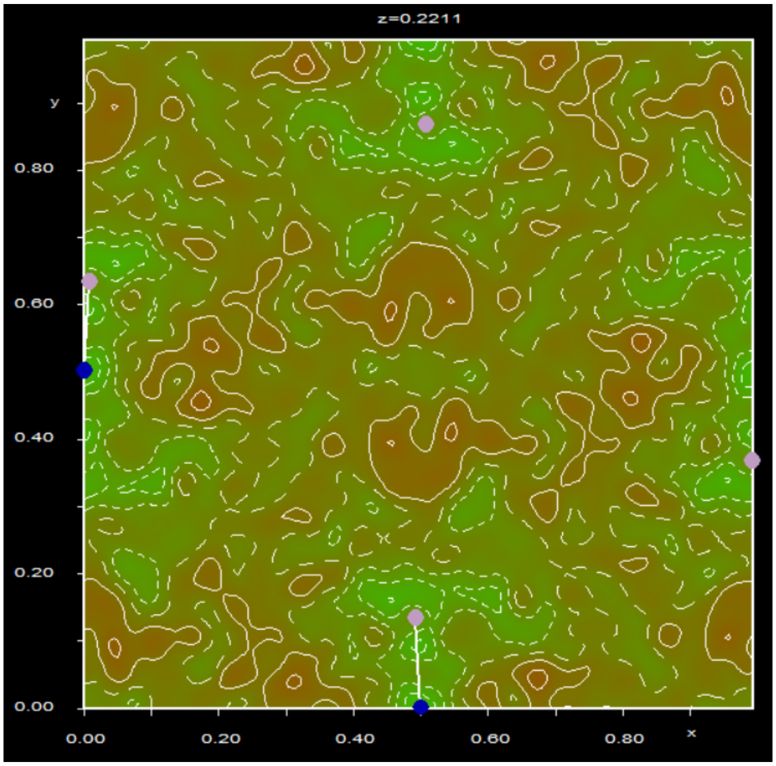

Figure S20. Difference map cross section calculated with the kinematical model of electron diffraction of the unit cell for **CoH**, oriented in the *xy* plane and cut at $z = 0.2211$. Any maxima/minima are too far from cobalt to be assigned as a hydride. Cobalt atoms are shown in blue, and phosphorus atoms are drawn in light purple.

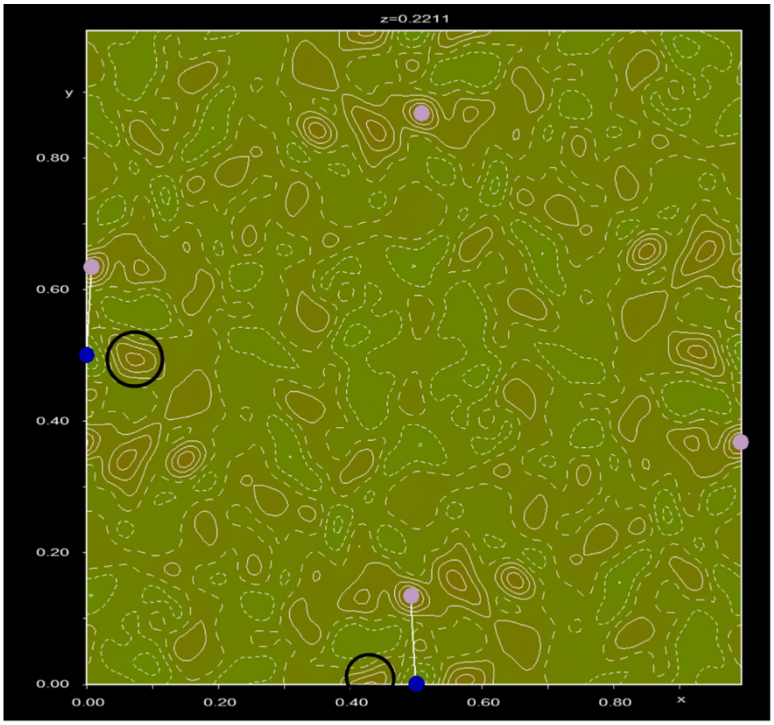

Figure S21. Difference map cross section calculated with the dynamical model of electron diffraction of the unit cell for **CoH**, oriented in the *xy* plane and cut at z = 0.2211. Density corresponding to the hydride is circled. Cobalt atoms are shown in blue, and phosphorus atoms are drawn in light purple.

Table S5. Experimental Details for Electron Diffraction of **CoH**.

Identification code	ED-25129-RSD-IV-17_16	
Empirical formula	$C_{24}H_{44}P_2Co$	
Formula weight	453.46	
Temperature	96(2) K	
Radiation type	Electron, λ = 0.0251 Å	
Crystal system	Tetragonal	
Space group	$I4_1cd$	
Unit cell dimensions	a = 16.2264(11) Å	α = 90°
	b = 16.2264(11) Å	β = 90°
	c = 19.0380(13) Å	γ = 90.
Volume	5012.6(6) $Å^3$	
Z	8	
Density (calculated)	1.202 g/cm^3	
F(000)	811	
Crystal size	0.0011 x 0.0003 x 0.0002 mm	
Crystal color and habit	triangular plate	
Diffractometer	Rigaku Synergy-ED, HyPix-ED	
Theta range for data collection	0.09, 0.9	
Index ranges	-20<=h<=20, -16<=k<=16, -22<=l<=22	
Reflections collected	11590	
Independent reflections	6745 [R(int) = 14.7 %)	
Observed reflections (I > 2sigma(I))	3634	
Completeness to theta = 29.69°	90 %	
Absorption correction	Semi-empirical from equivalents	
Solution method	SHELXT 2018/2 (Sheldrick, 2018)	
Refinement method	SHELXL 2019/3 (Sheldrick, 2015), Dynamical Jana2020 (Palatinus, 2023)	
Data / restraints / parameters	6745 / 0 / 140	
Goodness-of-fit on F^2	1.722	
Final R indices [I>2sigma(I)]	R1 = 17.1 %, wR2 = 40.9 %	
R indices (all data)	R1 = 19.9 %, wR2 = 42.9 %	
Largest diff. peak and hole	2.69 and –0.64 e $Å^{-3}$	

Table S6. MicroED datasets evaluated for refinement. Any dataset that was > 60 % complete and had a diffraction limit > 1.50 Å was refined. A refinement is considered stable if the hydride can be localized and has a positive, definite isotropic thermal parameter after refining with anisotropic thermal parameters on all non-hydrogen atoms.

Dataset	Completeness	Rint	Diffraction limit	Hydride localized	Stable refinement?
ED-25112-6	89.3 %	0.28	1.11	No	N/A
ED-25115-1	62.9 %	0.20	1.05	No	N/A
ED-25115-3	87.4 %	0.28	1.05	No	N/A
ED-25115-9	96.4 %	0.35	1.47	No	N/A
ED-25115-12	64.0 %	0.35	1.43	No	N/A
ED-25115-13	98.1 %	0.33	1.06	No	N/A
ED-25115-18	82.9 %	0.35	1.43	No	N/A
ED-25115-19	61.0 %	0.29	1.19	No	N/A
ED-25115-20	75.6 %	0.23	1.09	No	N/A
ED-25129-2	95.7 %	0.20	1.08	No	N/A
ED-25129-4	85.2 %	0.30	1.36	No	N/A
ED-25129-8	93.1 %	0.19	1.07	No	N/A
ED-25129-10	81.2 %	0.25	1.06	No	N/A
ED-25129-12	87.6 %	0.19	1.28	No	N/A
ED-25129-15	99.1 %	0.20	1.03	No	N/A
ED-25129-16	89.8 %	0.17	0.91	Yes	Yes
ED-25129-18	73.5 %	0.19	0.98	Yes	No
ED-25129-19	95.4 %	0.24	1.05	No	N/A
ED-25129-20	75.9 %	0.31	1.42	No	N/A

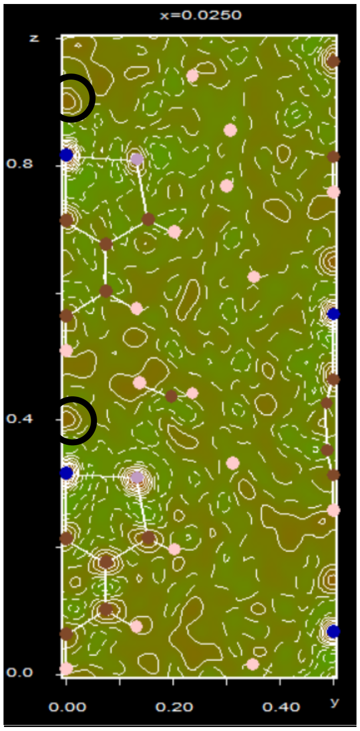

Figure S22. Difference map cross section calculated with the dynamical model of electron diffraction of the unit cell for ED-25129-18, oriented in the *xy* plane and cut at $z = 0.025$. Density corresponding to the hydride is circled. Cobalt atoms are shown in blue, and phosphorus atoms are drawn in light purple.

<u>*XYZ Coordinates from DFT Optimizations*</u>
PBE

Co	-8.06695	0.00000	8.32240
P	-7.94707	2.15791	8.48995
C	-7.83769	1.18745	11.03673
C	-6.93274	3.17031	6.05973
H	-7.55067	3.88827	5.96196
H	-6.14702	3.46879	5.61690
H	-7.27639	2.25875	5.67441
C	-9.53514	4.54653	8.32758
H	-9.01885	4.95311	7.61061
H	-10.50317	4.92084	8.26240
H	-9.13179	4.85630	9.16340
C	-8.06695	0.00000	13.12782

H	-8.06695	0.00000	13.99432
C	-9.59838	3.01672	8.23556
C	-10.52414	2.46687	9.33785
H	-10.21276	2.75890	10.27528
H	-11.39053	2.79116	9.25925
H	-10.59997	1.51659	9.29759
C	-6.56601	2.99832	7.54351
C	-10.18856	2.59917	6.86872
H	-10.19662	1.62952	6.80546
H	-11.05172	2.98477	6.74795
H	-9.64807	2.96864	6.05781
C	-6.12765	4.35131	8.11670
H	-5.77594	4.22708	9.08672
H	-5.34032	4.67883	7.66812
H	-6.79237	4.93697	8.08987
C	-8.06695	0.00000	10.28870
C	-7.51356	2.45881	10.27240
H	-7.98628	3.30745	10.62035
H	-6.55036	2.62983	10.35196
C	-5.38711	2.00060	7.62594
H	-5.61460	1.12937	7.20803
H	-4.63043	2.38782	7.20803
H	-5.14671	1.83926	8.56912
C	-7.85882	1.18423	12.43673
H	-7.69587	1.98447	12.93995
H	-8.06695	0.00000	6.86297
P	-8.18683	-2.15791	8.48995
C	-8.29621	-1.18745	11.03673
C	-9.20116	-3.17031	6.05973
H	-8.58324	-3.88827	5.96196

H	-9.98688	-3.46879	5.61690
H	-8.85751	-2.25875	5.67441
C	-6.59877	-4.54653	8.32758
H	-7.11505	-4.95311	7.61061
H	-5.63073	-4.92084	8.26240
H	-7.00211	-4.85630	9.16340
C	-6.53552	-3.01672	8.23556
C	-5.60976	-2.46687	9.33785
H	-5.92114	-2.75890	10.27528
H	-4.74337	-2.79116	9.25925
H	-5.53393	-1.51659	9.29759
C	-9.56789	-2.99832	7.54351
C	-5.94534	-2.59917	6.86872
H	-5.93727	-1.62952	6.80546
H	-5.08218	-2.98477	6.74795
H	-6.48583	-2.96864	6.05781
C	-10.00624	-4.35131	8.11670
H	-10.35796	-4.22708	9.08672
H	-10.79358	-4.67883	7.66812
H	-9.34153	-4.93697	8.08987
C	-8.62034	-2.45881	10.27240
H	-8.14762	-3.30745	10.62035
H	-9.58354	-2.62983	10.35196
C	-10.74679	-2.00060	7.62594
H	-10.51930	-1.12937	7.20803
H	-11.50347	-2.38782	7.20803
H	-10.98719	-1.83926	8.56912
C	-8.27508	-1.18423	12.43673
H	-8.43803	-1.98447	12.93995

PBE0

Co	-8.06695	0.00000	8.32240
P	-7.94707	2.15791	8.48995
C	-7.83769	1.18745	11.03673
C	-6.93274	3.17031	6.05973
H	-7.55067	3.88827	5.96196
H	-6.14702	3.46879	5.61690
H	-7.27639	2.25875	5.67441
C	-9.53514	4.54653	8.32758
H	-9.01885	4.95311	7.61061
H	-10.50317	4.92084	8.26240
H	-9.13179	4.85630	9.16340
C	-8.06695	0.00000	13.12782
H	-8.06695	0.00000	13.99432
C	-9.59838	3.01672	8.23556
C	-10.52414	2.46687	9.33785
H	-10.21276	2.75890	10.27528
H	-11.39053	2.79116	9.25925
H	-10.59997	1.51659	9.29759
C	-6.56601	2.99832	7.54351
C	-10.18856	2.59917	6.86872
H	-10.19662	1.62952	6.80546
H	-11.05172	2.98477	6.74795
H	-9.64807	2.96864	6.05781
C	-6.12765	4.35131	8.11670
H	-5.77594	4.22708	9.08672
H	-5.34032	4.67883	7.66812
H	-6.79237	4.93697	8.08987
C	-8.06695	0.00000	10.28870
C	-7.51356	2.45881	10.27240
H	-7.98628	3.30745	10.62035

H	-6.55036	2.62983	10.35196
C	-5.38711	2.00060	7.62594
H	-5.61460	1.12937	7.20803
H	-4.63043	2.38782	7.20803
H	-5.14671	1.83926	8.56912
C	-7.85882	1.18423	12.43673
H	-7.69587	1.98447	12.93995
H	-8.06695	0.00000	6.86297
P	-8.18683	-2.15791	8.48995
C	-8.29621	-1.18745	11.03673
C	-9.20116	-3.17031	6.05973
H	-8.58324	-3.88827	5.96196
H	-9.98688	-3.46879	5.61690
H	-8.85751	-2.25875	5.67441
C	-6.59877	-4.54653	8.32758
H	-7.11505	-4.95311	7.61061
H	-5.63073	-4.92084	8.26240
H	-7.00211	-4.85630	9.16340
C	-6.53552	-3.01672	8.23556
C	-5.60976	-2.46687	9.33785
H	-5.92114	-2.75890	10.27528
H	-4.74337	-2.79116	9.25925
H	-5.53393	-1.51659	9.29759
C	-9.56789	-2.99832	7.54351
C	-5.94534	-2.59917	6.86872
H	-5.93727	-1.62952	6.80546
H	-5.08218	-2.98477	6.74795
H	-6.48583	-2.96864	6.05781
C	-10.00624	-4.35131	8.11670
H	-10.35796	-4.22708	9.08672

H	-10.79358	-4.67883	7.66812
H	-9.34153	-4.93697	8.08987
C	-8.62034	-2.45881	10.27240
H	-8.14762	-3.30745	10.62035
H	-9.58354	-2.62983	10.35196
C	-10.74679	-2.00060	7.62594
H	-10.51930	-1.12937	7.20803
H	-11.50347	-2.38782	7.20803
H	-10.98719	-1.83926	8.56912
C	-8.27508	-1.18423	12.43673
H	-8.43803	-1.98447	12.93995

BP86

Co	-8.06695	0.00000	8.32240
P	-7.94707	2.15791	8.48995
C	-7.83769	1.18745	11.03673
C	-6.93274	3.17031	6.05973
H	-7.55067	3.88827	5.96196
H	-6.14702	3.46879	5.61690
H	-7.27639	2.25875	5.67441
C	-9.53514	4.54653	8.32758
H	-9.01885	4.95311	7.61061
H	-10.50317	4.92084	8.26240
H	-9.13179	4.85630	9.16340
C	-8.06695	0.00000	13.12782
H	-8.06695	0.00000	13.99432
C	-9.59838	3.01672	8.23556
C	-10.52414	2.46687	9.33785
H	-10.21276	2.75890	10.27528
H	-11.39053	2.79116	9.25925
H	-10.59997	1.51659	9.29759

C	-6.56601	2.99832	7.54351
C	-10.18856	2.59917	6.86872
H	-10.19662	1.62952	6.80546
H	-11.05172	2.98477	6.74795
H	-9.64807	2.96864	6.05781
C	-6.12765	4.35131	8.11670
H	-5.77594	4.22708	9.08672
H	-5.34032	4.67883	7.66812
H	-6.79237	4.93697	8.08987
C	-8.06695	0.00000	10.28870
C	-7.51356	2.45881	10.27240
H	-7.98628	3.30745	10.62035
H	-6.55036	2.62983	10.35196
C	-5.38711	2.00060	7.62594
H	-5.61460	1.12937	7.20803
H	-4.63043	2.38782	7.20803
H	-5.14671	1.83926	8.56912
C	-7.85882	1.18423	12.43673
H	-7.69587	1.98447	12.93995
H	-8.06695	0.00000	6.86297
P	-8.18683	-2.15791	8.48995
C	-8.29621	-1.18745	11.03673
C	-9.20116	-3.17031	6.05973
H	-8.58324	-3.88827	5.96196
H	-9.98688	-3.46879	5.61690
H	-8.85751	-2.25875	5.67441
C	-6.59877	-4.54653	8.32758
H	-7.11505	-4.95311	7.61061
H	-5.63073	-4.92084	8.26240
H	-7.00211	-4.85630	9.16340

C	-6.53552	-3.01672	8.23556
C	-5.60976	-2.46687	9.33785
H	-5.92114	-2.75890	10.27528
H	-4.74337	-2.79116	9.25925
H	-5.53393	-1.51659	9.29759
C	-9.56789	-2.99832	7.54351
C	-5.94534	-2.59917	6.86872
H	-5.93727	-1.62952	6.80546
H	-5.08218	-2.98477	6.74795
H	-6.48583	-2.96864	6.05781
C	-10.00624	-4.35131	8.11670
H	-10.35796	-4.22708	9.08672
H	-10.79358	-4.67883	7.66812
H	-9.34153	-4.93697	8.08987
C	-8.62034	-2.45881	10.27240
H	-8.14762	-3.30745	10.62035
H	-9.58354	-2.62983	10.35196
C	-10.74679	-2.00060	7.62594
H	-10.51930	-1.12937	7.20803
H	-11.50347	-2.38782	7.20803
H	-10.98719	-1.83926	8.56912
C	-8.27508	-1.18423	12.43673
H	-8.43803	-1.98447	12.93995

B3LYP

Co	-8.06695	0.00000	8.32240
P	-7.94707	2.15791	8.48995
C	-7.83769	1.18745	11.03673
C	-6.93274	3.17031	6.05973
H	-7.55067	3.88827	5.96196
H	-6.14702	3.46879	5.61690

H	-7.27639	2.25875	5.67441
C	-9.53514	4.54653	8.32758
H	-9.01885	4.95311	7.61061
H	-10.50317	4.92084	8.26240
H	-9.13179	4.85630	9.16340
C	-8.06695	0.00000	13.12782
H	-8.06695	0.00000	13.99432
C	-9.59838	3.01672	8.23556
C	-10.52414	2.46687	9.33785
H	-10.21276	2.75890	10.27528
H	-11.39053	2.79116	9.25925
H	-10.59997	1.51659	9.29759
C	-6.56601	2.99832	7.54351
C	-10.18856	2.59917	6.86872
H	-10.19662	1.62952	6.80546
H	-11.05172	2.98477	6.74795
H	-9.64807	2.96864	6.05781
C	-6.12765	4.35131	8.11670
H	-5.77594	4.22708	9.08672
H	-5.34032	4.67883	7.66812
H	-6.79237	4.93697	8.08987
C	-8.06695	0.00000	10.28870
C	-7.51356	2.45881	10.27240
H	-7.98628	3.30745	10.62035
H	-6.55036	2.62983	10.35196
C	-5.38711	2.00060	7.62594
H	-5.61460	1.12937	7.20803
H	-4.63043	2.38782	7.20803
H	-5.14671	1.83926	8.56912
C	-7.85882	1.18423	12.43673

H	-7.69587	1.98447	12.93995
H	-8.06695	0.00000	6.86297
P	-8.18683	-2.15791	8.48995
C	-8.29621	-1.18745	11.03673
C	-9.20116	-3.17031	6.05973
H	-8.58324	-3.88827	5.96196
H	-9.98688	-3.46879	5.61690
H	-8.85751	-2.25875	5.67441
C	-6.59877	-4.54653	8.32758
H	-7.11505	-4.95311	7.61061
H	-5.63073	-4.92084	8.26240
H	-7.00211	-4.85630	9.16340
C	-6.53552	-3.01672	8.23556
C	-5.60976	-2.46687	9.33785
H	-5.92114	-2.75890	10.27528
H	-4.74337	-2.79116	9.25925
H	-5.53393	-1.51659	9.29759
C	-9.56789	-2.99832	7.54351
C	-5.94534	-2.59917	6.86872
H	-5.93727	-1.62952	6.80546
H	-5.08218	-2.98477	6.74795
H	-6.48583	-2.96864	6.05781
C	-10.00624	-4.35131	8.11670
H	-10.35796	-4.22708	9.08672
H	-10.79358	-4.67883	7.66812
H	-9.34153	-4.93697	8.08987
C	-8.62034	-2.45881	10.27240
H	-8.14762	-3.30745	10.62035
H	-9.58354	-2.62983	10.35196
C	-10.74679	-2.00060	7.62594

H	-10.51930	-1.12937	7.20803
H	-11.50347	-2.38782	7.20803
H	-10.98719	-1.83926	8.56912
C	-8.27508	-1.18423	12.43673
H	-8.43803	-1.98447	12.93995

M06-2X

Co	-8.06695	0.00000	8.32240
P	-7.94707	2.15791	8.48995
C	-7.83769	1.18745	11.03673
C	-6.93274	3.17031	6.05973
H	-7.55067	3.88827	5.96196
H	-6.14702	3.46879	5.61690
H	-7.27639	2.25875	5.67441
C	-9.53514	4.54653	8.32758
H	-9.01885	4.95311	7.61061
H	-10.50317	4.92084	8.26240
H	-9.13179	4.85630	9.16340
C	-8.06695	0.00000	13.12782
H	-8.06695	0.00000	13.99432
C	-9.59838	3.01672	8.23556
C	-10.52414	2.46687	9.33785
H	-10.21276	2.75890	10.27528
H	-11.39053	2.79116	9.25925
H	-10.59997	1.51659	9.29759
C	-6.56601	2.99832	7.54351
C	-10.18856	2.59917	6.86872
H	-10.19662	1.62952	6.80546
H	-11.05172	2.98477	6.74795
H	-9.64807	2.96864	6.05781
C	-6.12765	4.35131	8.11670

H	-5.77594	4.22708	9.08672
H	-5.34032	4.67883	7.66812
H	-6.79237	4.93697	8.08987
C	-8.06695	0.00000	10.28870
C	-7.51356	2.45881	10.27240
H	-7.98628	3.30745	10.62035
H	-6.55036	2.62983	10.35196
C	-5.38711	2.00060	7.62594
H	-5.61460	1.12937	7.20803
H	-4.63043	2.38782	7.20803
H	-5.14671	1.83926	8.56912
C	-7.85882	1.18423	12.43673
H	-7.69587	1.98447	12.93995
H	-8.06695	0.00000	6.86297
P	-8.18683	-2.15791	8.48995
C	-8.29621	-1.18745	11.03673
C	-9.20116	-3.17031	6.05973
H	-8.58324	-3.88827	5.96196
H	-9.98688	-3.46879	5.61690
H	-8.85751	-2.25875	5.67441
C	-6.59877	-4.54653	8.32758
H	-7.11505	-4.95311	7.61061
H	-5.63073	-4.92084	8.26240
H	-7.00211	-4.85630	9.16340
C	-6.53552	-3.01672	8.23556
C	-5.60976	-2.46687	9.33785
H	-5.92114	-2.75890	10.27528
H	-4.74337	-2.79116	9.25925
H	-5.53393	-1.51659	9.29759
C	-9.56789	-2.99832	7.54351

C	-5.94534	-2.59917	6.86872
H	-5.93727	-1.62952	6.80546
H	-5.08218	-2.98477	6.74795
H	-6.48583	-2.96864	6.05781
C	-10.00624	-4.35131	8.11670
H	-10.35796	-4.22708	9.08672
H	-10.79358	-4.67883	7.66812
H	-9.34153	-4.93697	8.08987
C	-8.62034	-2.45881	10.27240
H	-8.14762	-3.30745	10.62035
H	-9.58354	-2.62983	10.35196
C	-10.74679	-2.00060	7.62594
H	-10.51930	-1.12937	7.20803
H	-11.50347	-2.38782	7.20803
H	-10.98719	-1.83926	8.56912
C	-8.27508	-1.18423	12.43673
H	-8.43803	-1.98447	12.93995

References

(1) Macrae, C. F.; Sovago, I.; Cottrell, S. J.; Galek, P. T. A.; McCabe, P.; Pidcock, E.; Platings, M.; Shields, G. P.; Stevens, J. S.; Towler, M.; Wood, P. A. Mercury 4.0: From Visualization to Analysis, Design and Prediction. *J. Appl. Cryst.* **2020**, *53* (1), 226–235. https://doi.org/10.1107/S1600576719014092.
(2) Momma, K.; Izumi, F. VESTA 3 for Three-Dimensional Visualization of Crystal, Volumetric and Morphology Data. *J. Appl. Cryst.* **2011**, *44* (6), 1272–1276. https://doi.org/10.1107/S0021889811038970.
(3) Kuriyama, S.; Wei, S.; Tanaka, H.; Konomi, A.; Yoshizawa, K.; Nishibayashi, Y. Synthesis and Reactivity of Cobalt–Dinitrogen Complexes Bearing Anionic PCP-Type Pincer Ligands toward Catalytic Silylamine Formation from Dinitrogen. *Inorg. Chem.* **2022**, *61* (13), 5190–5195. https://doi.org/10.1021/acs.inorgchem.2c00234.
(4) Stoll, S.; Schweiger, A. EasySpin, a Comprehensive Software Package for Spectral Simulation and Analysis in EPR. *J. Mag. Res.* **2006**, *178* (1), 42–55. https://doi.org/10.1016/j.jmr.2005.08.013.
(5) Bain, G. A.; Berry, J. F. Diamagnetic Corrections and Pascal's Constants. *J. Chem. Educ.* **2008**, *85* (4), 532. https://doi.org/10.1021/ed085p532.
(6) Azuah, R. T.; Kneller, L. R.; Qiu, Y.; Tregenna-Piggott, P. L. W.; Brown, C. M.; Copley, J. R. D.; Dimeo, R. M. DAVE: A Comprehensive Software Suite for the Reduction, Visualization, and Analysis of Low Energy Neutron Spectroscopic Data. *J. Res. Natl. Inst. Stand. Technol.* **2009**, *114* (6), 341. https://doi.org/10.6028/jres.114.025.
(7) CrysAlisPro; Rigaku OD: The Woodlands, TX, 2015.
(8) Sheldrick, G. M. SHELXT – Integrated Space-Group and Crystal-Structure Determination. *Acta Cryst. A* **2015**, *71* (1), 3–8. https://doi.org/10.1107/S2053273314026370.
(9) Sheldrick, G. M. Crystal Structure Refinement with SHELXL. *Acta Cryst. C* **2015**, *71* (1), 3–8. https://doi.org/10.1107/S2053229614024218.
(10) Dolomanov, O. V.; Bourhis, L. J.; Gildea, R. J.; Howard, J. a. K.; Puschmann, H. OLEX2: A Complete Structure Solution, Refinement and Analysis Program. *J. Appl. Cryst.* **2009**, *42* (2), 339–341. https://doi.org/10.1107/S0021889808042726.
(11) Groom, C. R.; Bruno, I. J.; Lightfoot, M. P.; Ward, S. C. The Cambridge Structural Database. *Acta Cryst. B* **2016**, *72* (2), 171–179. https://doi.org/10.1107/S2052520616003954.
(12) Kleemiss, F.; Dolomanov, O. V.; Bodensteiner, M.; Peyerimhoff, N.; Midgley, L.; Bourhis, L. J.; Genoni, A.; Malaspina, L. A.; Jayatilaka, D.; Spencer, J. L.; White, F.; Grundkötter-Stock, B.; Steinhauer, S.; Lentz, D.; Puschmann, H.; Grabowsky, S. Accurate Crystal Structures and Chemical Properties from NoSpherA2. *Chem. Sci.* **2021**, *12* (5), 1675–1692. https://doi.org/10.1039/D0SC05526C.
(13) Neese, F. Software Update: The ORCA Program System—Version 6.0. *WIREs Computational Molecular Science* **2025**, *15* (2), e70019. https://doi.org/10.1002/wcms.70019.
(14) *Enhancing Neutron Single Crystal Visualization With NeuXtalViz*. https://www.programmaster.org/PM/PM.nsf/ApprovedAbstracts/ED26ABC7E89A1A5F85258BD40061D3B7?OpenDocument (accessed 2026-02-24).

(15) Reshniak, V.; Wang, X.; Zhang, G.; Liu, S.; Yin, J. Hierarchical Bayesian Approach for Adaptive Integration of Bragg Peaks in Time-of-Flight Neutron Scattering Data. arXiv April 1, 2024. https://doi.org/10.48550/arXiv.2406.05133.
(16) Schultz, A. J.; Jørgensen, M. R. V.; Wang, X.; Mikkelson, R. L.; Mikkelson, D. J.; Lynch, V. E.; Peterson, P. F.; Green, M. L.; Hoffmann, C. M. Integration of Neutron Time-of-Flight Single-Crystal Bragg Peaks in Reciprocal Space. *J. Appl. Cryst.* **2014**, *47* (3), 915–921. https://doi.org/10.1107/S1600576714006372.
(17) Petříček, V.; Palatinus, L.; Plášil, J.; Dušek, M. Jana2020 – a New Version of the Crystallographic Computing System Jana. *Z. für Krist. Cryst. Mat.* **2023**, *238* (7–8), 271–282. https://doi.org/10.1515/zkri-2023-0005.
